# Supplementary material for: 3D microprinting of inorganic porous materials by chemical linking-induced solidification of nanocrystals
Source: Nat Commun. 2023 Dec 20;14:8460. doi: 10.1038/s41467-023-44145-7 (PMC10733400; doi:10.1038/s41467-023-44145-7)
Supplement: Supplementary file 1 — Supplementary Information [file 41467_2023_44145_MOESM1_ESM.pdf]

Supplementary Information

3D microprinting of inorganic porous materials by chemical linking-induced solidification of nanocrystals

Minju Song[1], Yoonkyum Kim[1], Du San Baek[2], Ho Young Kim[3], Da Hwi Gu[1], Haiyang Li[4], Benjamin V. Cunning[5], Seong Eun Yang[1], Seung Hwae Heo[4], Seunghyun Lee[1], Minhyuk Kim[6], June Sung Lim[7,8], Hu Young Jeong[6], Jung-Woo Yoo[1], Sang Hoon Joo[8], Rodney S. Ruoff[5,2,1], Jin Young Kim[3*], and Jae Sung Son[4*]

[1]*Department of Materials Science and Engineering, Ulsan National Institute of Science and Technology (UNIST), Ulsan 44919, Republic of Korea*

[2]*Department of Chemistry, Ulsan National Institute of Science and Technology (UNIST), Ulsan 44919, Republic of Korea*

[3]*Hydrogen·Fuel Cell Research Center, Korea Institute of Science and Technology (KIST), 14-gil 5 Hwarang-ro, Seongbuk-gu, Seoul 02792, Republic of Korea*

[4]*Department of Chemical Engineering, Pohang University of Science and Technology (POSTECH), Gyeongsangbuk-do, 37673 Republic of Korea.*

[5]*Center for Multidimensional Carbon Materials (CMCM), Institute for Basic Science (IBS), Ulsan 44919, Republic of Korea.*

[6]*Graduate School of Semiconductor Materials and Devices Engineering, Ulsan National Institute of Science and Technology (UNIST), Ulsan 44919, Republic of Korea.*

[7]*School of Energy and Chemical Engineering, Ulsan National Institute of Science and Technology (UNIST), Ulsan 44919, Republic of Korea.*

[8]*Department of Chemistry, Seoul National University, Seoul 08826, Republic of Korea.*

*Correspondence to: jinykim@kist.re.kr (J.Y.K.) & sonjs@postech.ac.kr (J.S.S.)

This file includes:

Supplementary Fig. S1–S31 partially with explanatory text

Supplementary Table 1–11

Supplementary discussion

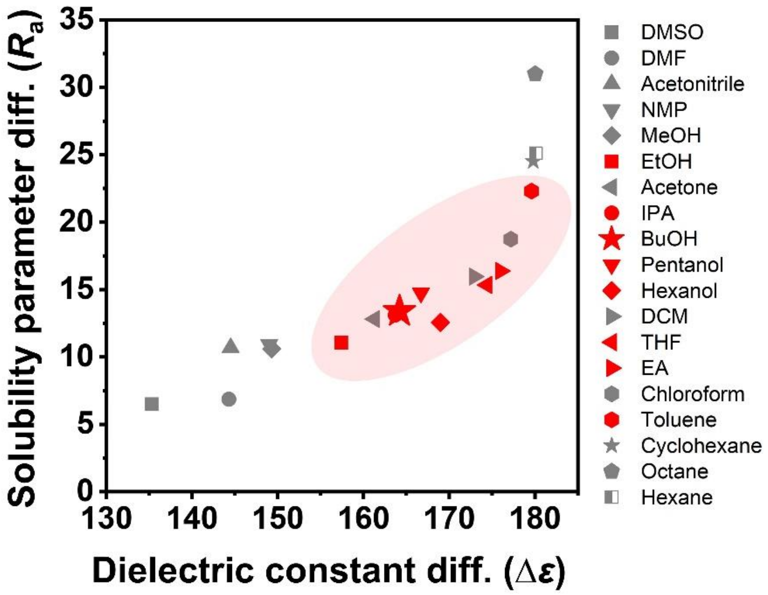

Supplementary Fig. 1 | Hansen solubility parameter (HSP) difference (*R*$_a$) versus dielectric constant difference ($\Delta\varepsilon$) between NMF (ε: 182) and various nonsolvents. The grey and red symbols represent the non-printable and printable NMF-nonsolvent combinations, respectively (see the detailed data and symbol description in Supplementary Fig. 2 and Supplementary Table 1, respectively). Hereafter, NMF-BuOH was chosen as the solvent-nonsolvent combination for printing.

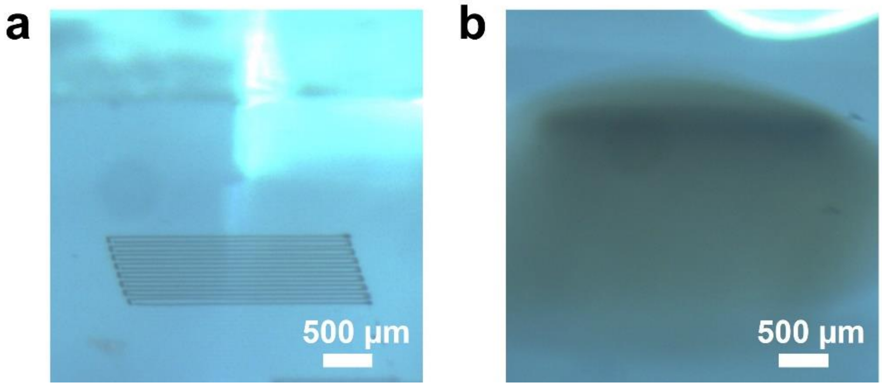

Supplementary Fig. 2 | Comparison of Ag nanocrystal ink printed in linker-ion-containing bath with nonsolvent and solvent. a,b, CCD camera images of printed Ag nanocrystal ink in linker-ion-containing BuOH (**a**) and NMF (**b**) baths, respectively.

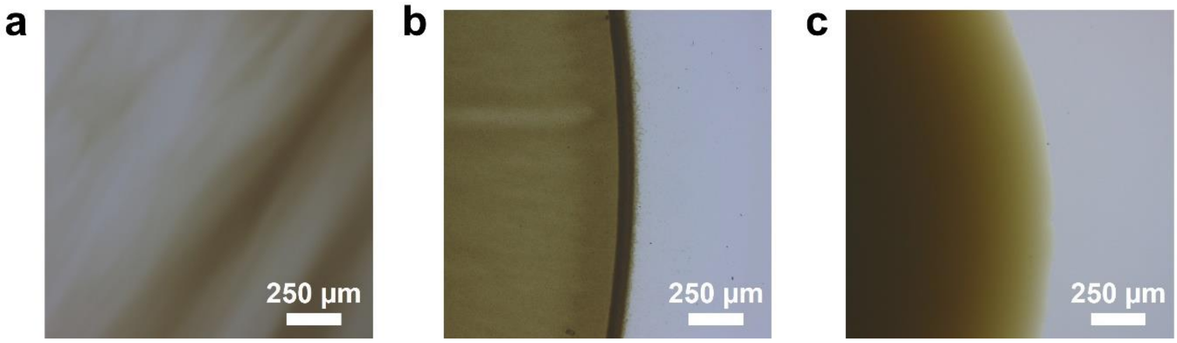

Supplementary Fig. 3 | OM images of non-printable and printable NMF-nonsolvent combinations. a–c, Ag nanocrystal ink printed in DMSO (**a**), BuOH (**b**), and hexane (**c**) representing non-printable (miscible), printable, and non-printable (immiscible), respectively.

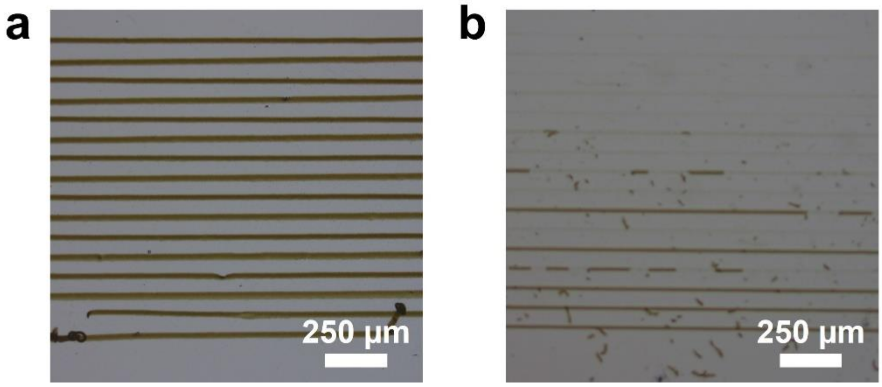

Supplementary Fig. 4 | Comparison of Ag nanocrystal ink printed in solidification bath with and without linker ions. **a,b,** Optical microscope images of printed Ag nanocrystal ink in solidification bath with (**a**) and without (**b**) linker ions, respectively after harsh shaking.

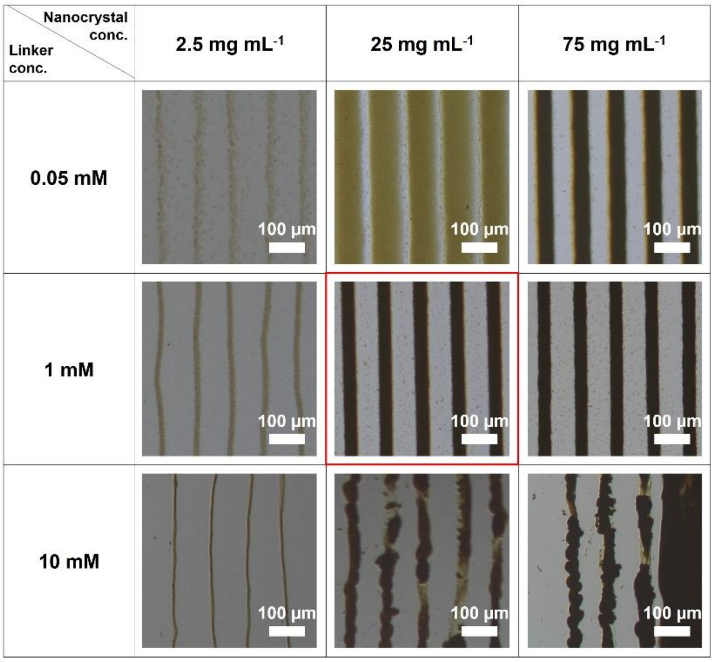

Supplementary Fig. 5 | Optimisation of linking of nanocrystals with the concentration of linker and nanocrystal. OM images showing Ag nanocrystal filaments printed in the Au^{3+} linker bath with control parameters of linker and nanocrystal ink concentrations.

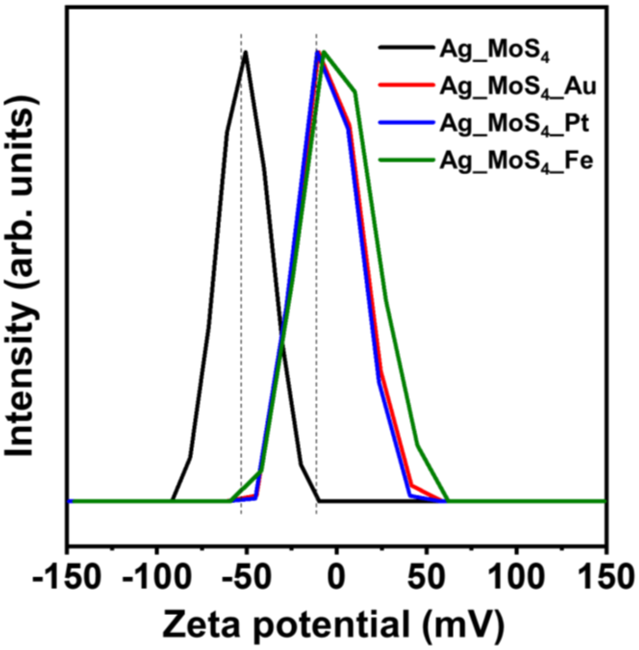

Supplementary Fig. 6 | ζ-potentials of the wet state Ag printed with various metal ion linker molecules. ζ-potential of thiomolybdate-capped Ag nanocrystal inks before (black line) and after the exposure to linker baths with Au^{3+} (red), Pt^{4+} (blue), and Fe^{2+} (green) metal ions, respectively.

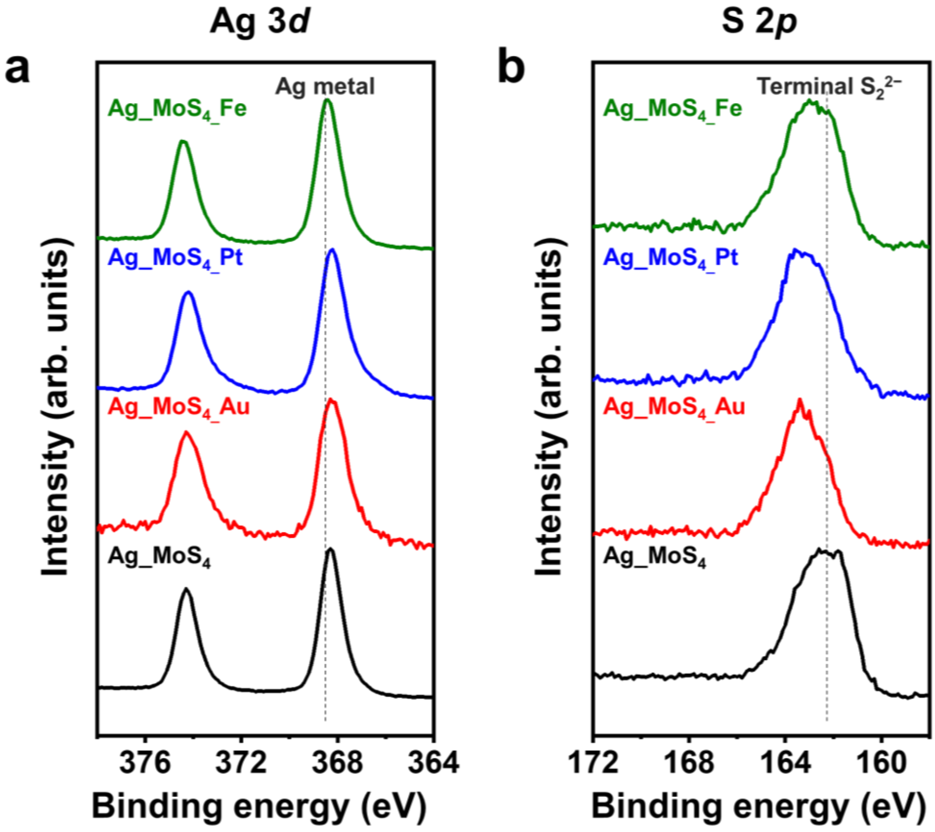

Supplementary Fig. 7 | XPS analysis of the Ag nanocrystals printed with various metal ion linker molecules. The printed Ag nanocrystals was measured after the supercritical CO_2 drying. **a,b,** Ag 3*d* (**a**) and S 2*p* (**b**) XPS spectra of thiomolybdate-capped Ag nanocrystal inks after exposure to linker containing solidification baths with no linker molecule (black), Au^{3+} (red), Pt^{4+} (blue), and Fe^{2+} (green) linker molecules, respectively. All XPS spectra were corrected with adventitious C 1s peak at 284.8 eV.

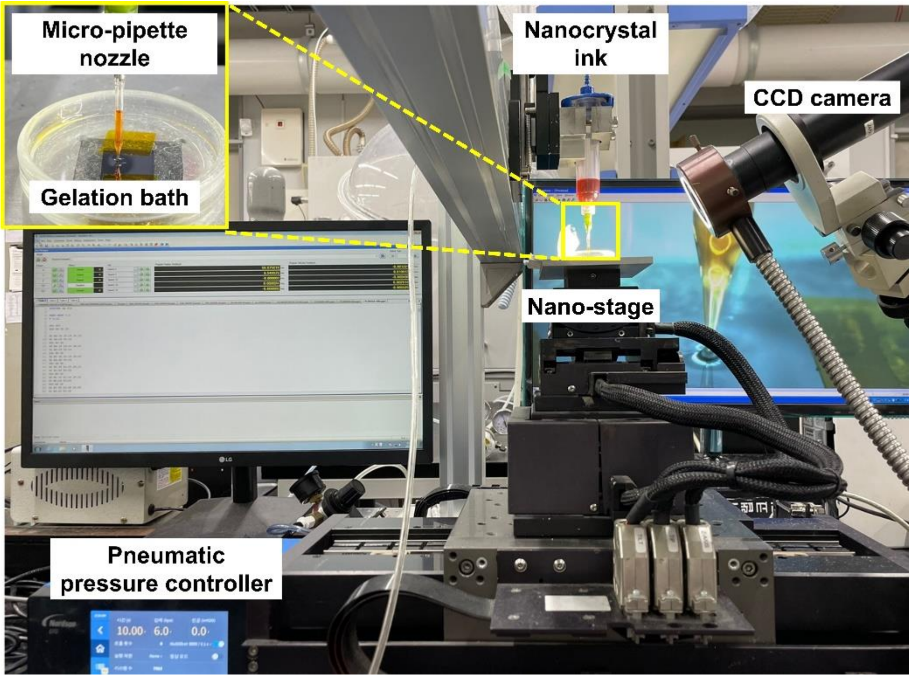

Supplementary Fig. 8 | 3D microprinting set-up. The photograph shows the experimental printing set-up comprising the moving stage along the x-, y-, and z-axes, pneumatic pressure controller, CCD camera, and nanocrystal ink. The inset shows the micro-pipette nozzle connected to a syringe-type reservoir, and linker containing solidification bath.

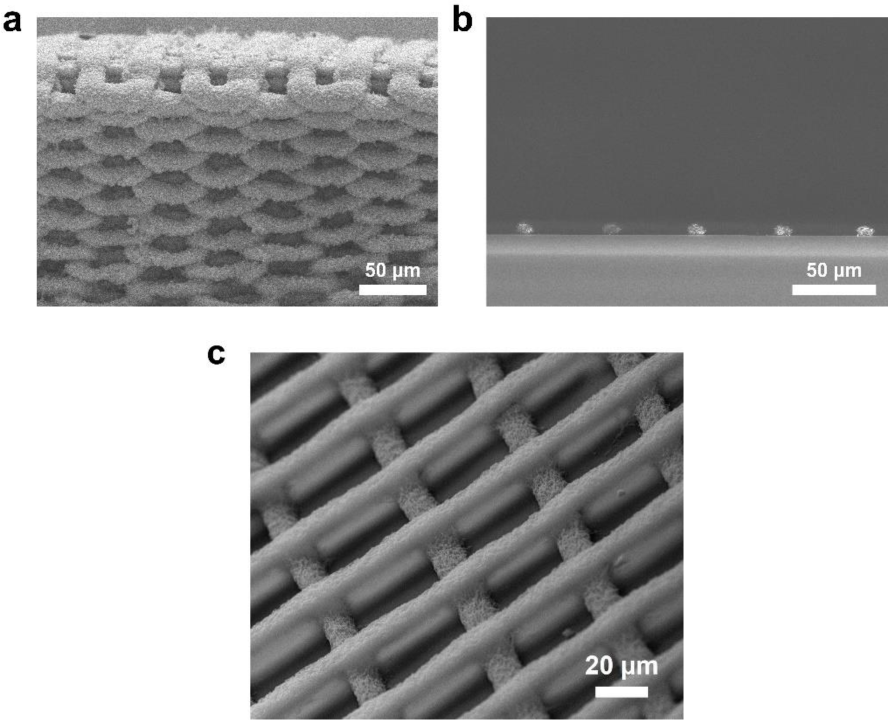

Supplementary Fig. 9 | Structural analysis of the printed objects. a, Side-view SEM image of the printed 3D lattice cube of printed Ag object. **b,** Cross-sectional SEM image of the printed Ag filaments. **c**, OM image of the printed Fe_3O_4 lattice.

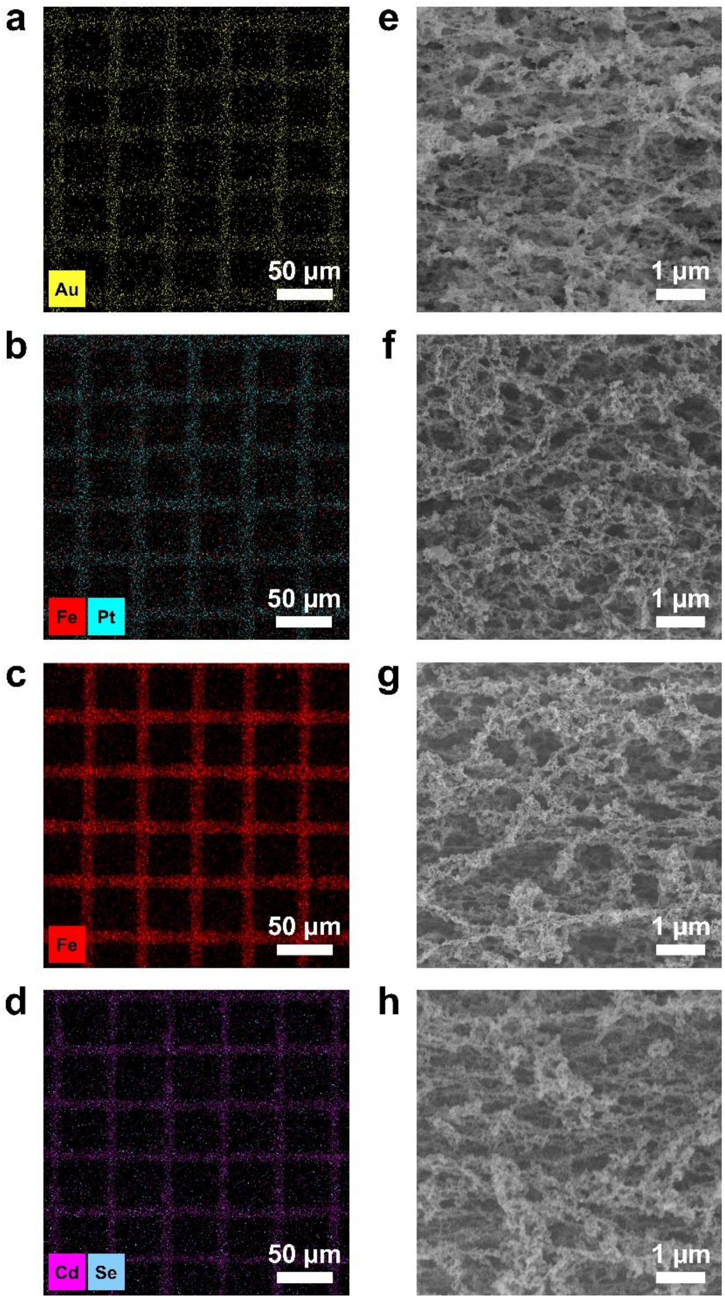

Supplementary Fig. 10 | Composition and microstructure analysis of printed inorganic nanocrystal-based porous materials. a–d, EDS mapping images of the printed 3D lattices of Au (**a**), FePt (**b**), Fe_3O_4 (**c**), and CdSe (**d**). **e–h,** HRSEM images of the printed 3D lattices of Au (**e**), FePt (**f**), Fe_3O_4 (**g**), and CdSe (**h**).

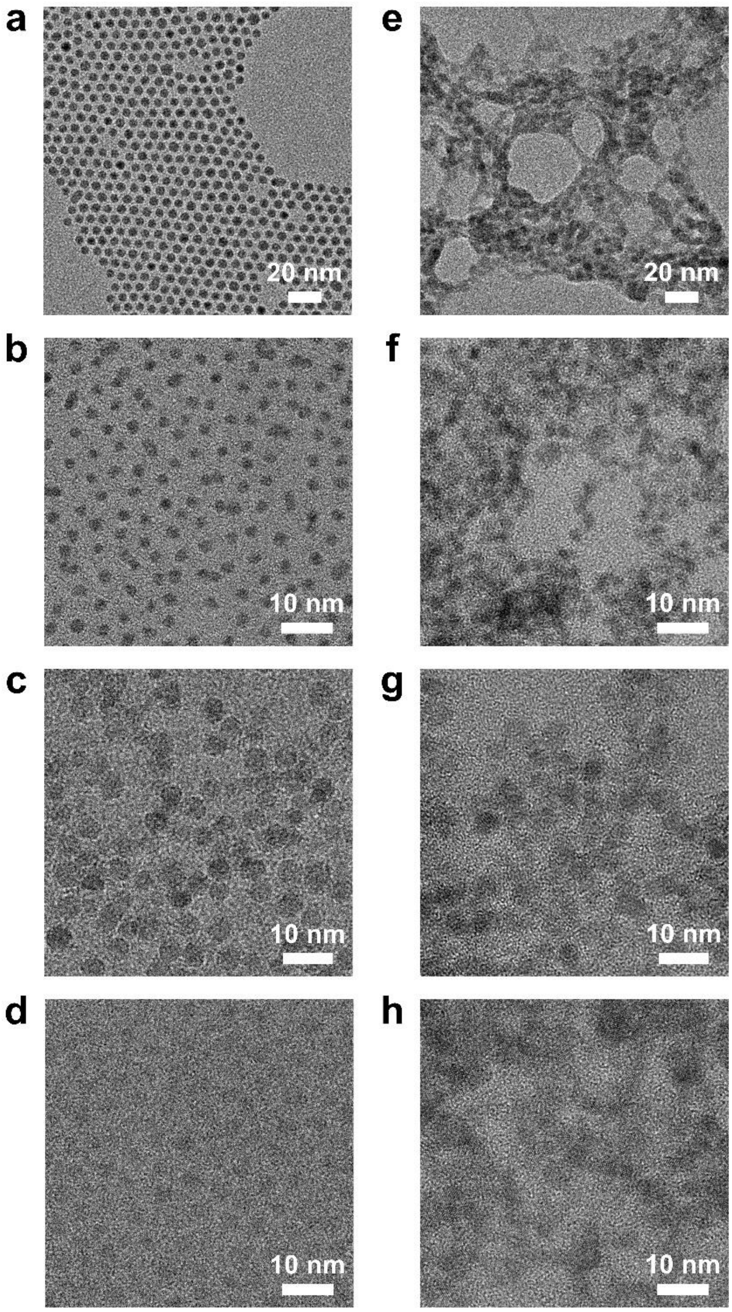

Supplementary Fig. 11 | TEM images of as-synthesised nanocrystals and printed nanocrystals. a–h, TEM images of as-synthesised Ag (**a**), FePt (**b**), Fe_3O_4 (**c**), and CdSe (**d**) nanocrystals and the corresponding printed nanocrystal-based porous materials (**e–h**). For the sample preparation of the printed nanocrystals, thiomolybdate-capped Ag (**e**), FePt (**f**), Fe_3O_4 (**g**), and CdSe (**h**) nanocrystals were linked with Au^{3+} (Ag), Pt^{4+} (FePt), and Fe^{2+} (Fe_3O_4 and CdSe), respectively.

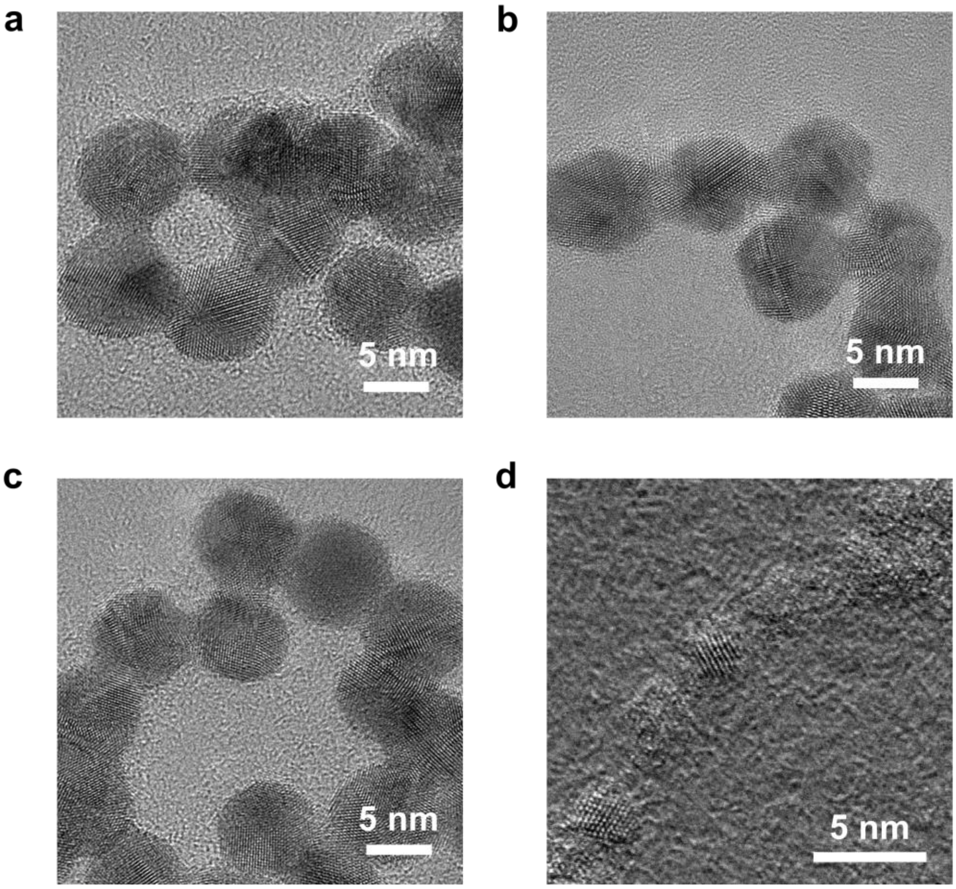

Supplementary Fig. 12 | High resolution TEM and HADDF-STEM images of the printed Au and FePt nanocrystals. a-d, HRTEM images of the printed thiomolybdate-capped Au nanocrystals (**a**-**c**) linked with Au^{3+} and thiomolybdate-capped FePt nanocrystals linked with Pt^{4+} (**d**).

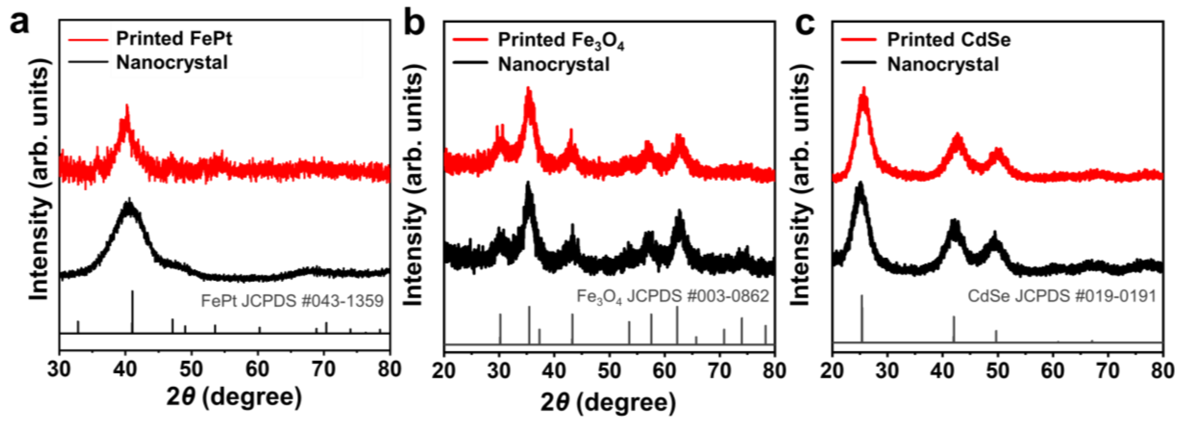

Supplementary Fig. 13 | XRD analysis of as-synthesised nanocrystals and printed nanocrystal-based porous materials. **a–c,** XRD patterns of as-synthesised FePt (**a**), Fe_3O_4 (**b**), and CdSe (**c**) nanocrystals and the corresponding printed nanocrystal-based porous materials.

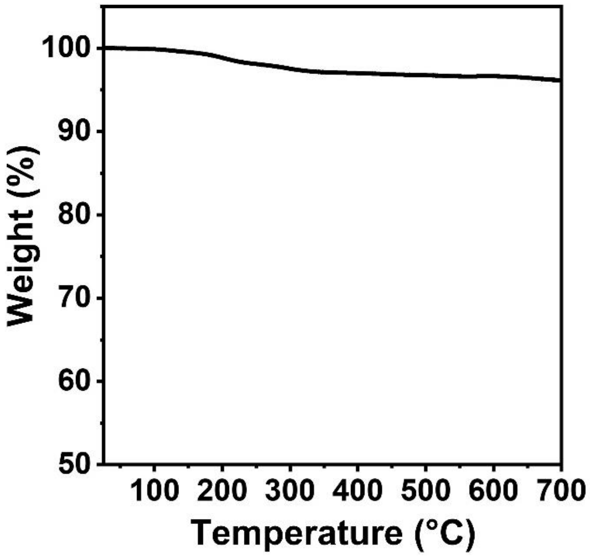

Supplementary Fig. 14 | Thermogravimetric scans of the printed Au.

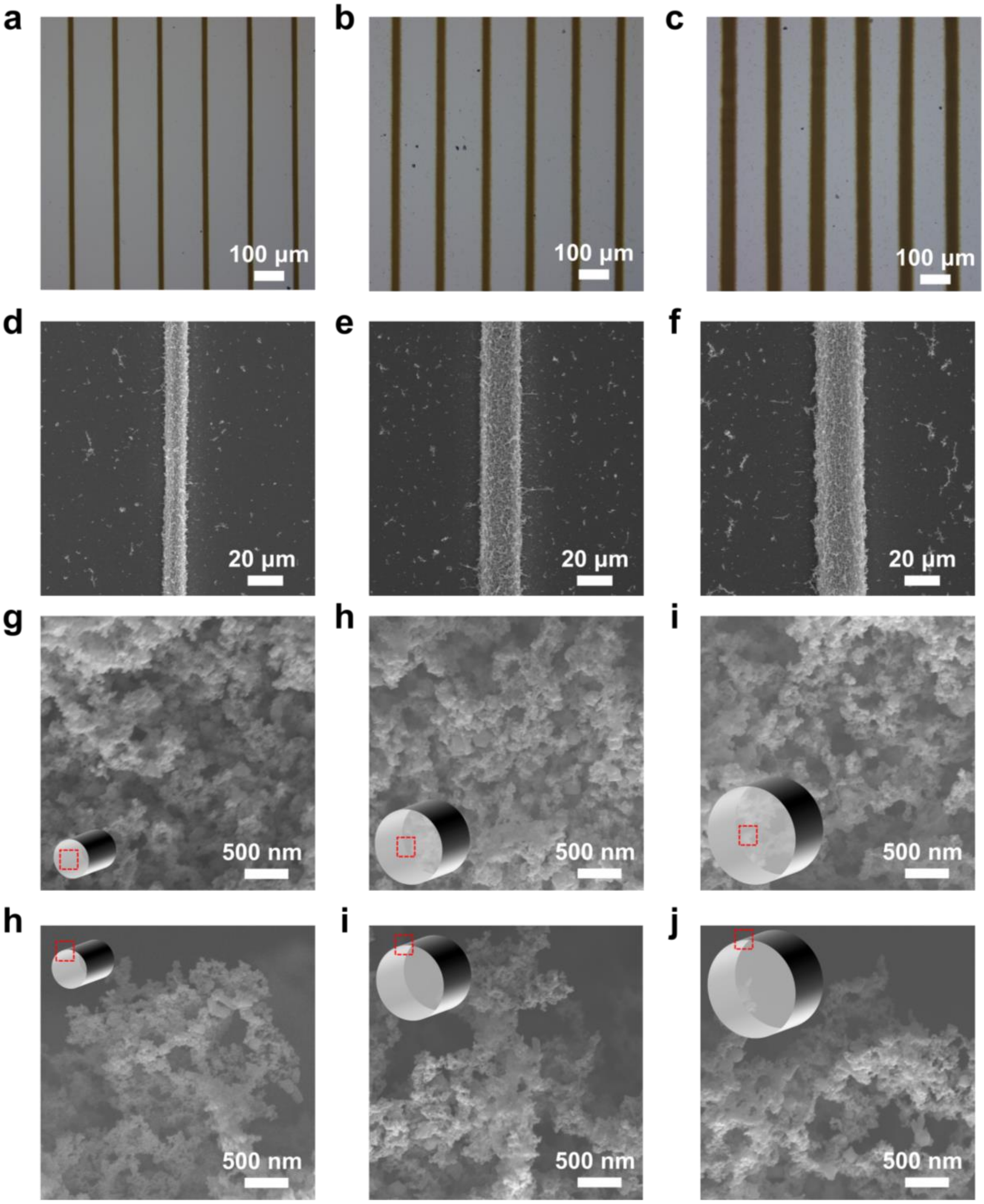

Supplementary Fig. 15 | Microstructural analysis on the printed filament with different diameters. OM images (a-c), low-magnification SEM images (d-f), high-magnification cross-sectional SEM of interior (g-i) and exterior surface (h-j) of the Ag printed filaments with the diameters of 12, 23, and 30 μm, respectively. The insets in the panels g-i indicate the analysed regions of the printed filaments.

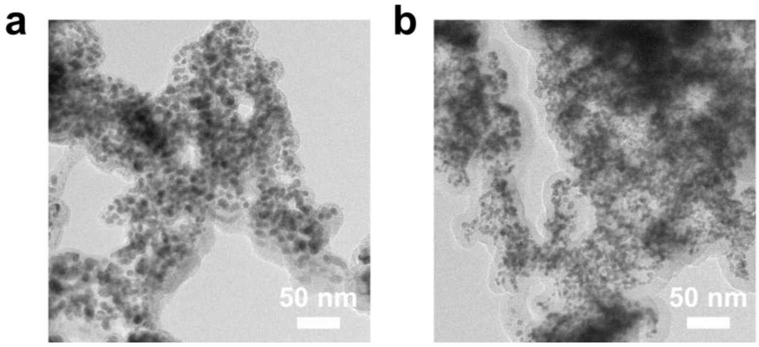

Supplementary Fig. 16 | TEM analysis on the Ag printed filament. Cross-sectional TEM images of interior (**a**) and exterior surface (**b**) of the Ag filament printed with the diameters of 12 μm.

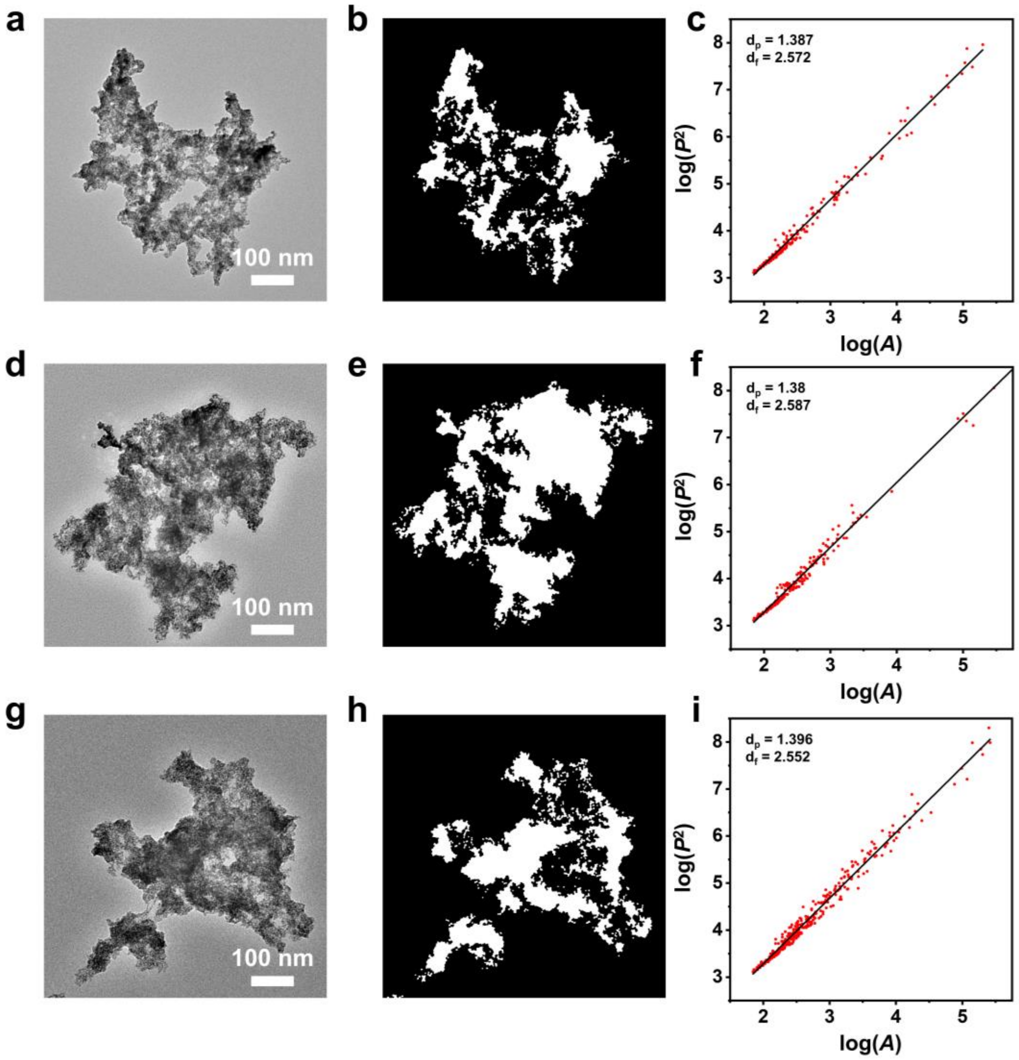

Supplementary Fig. 17 | Calculation of d_f of the printed Ag filaments. a,d,g, TEM images of the fractal structures in the Ag printed filaments with the diameters of 12, 23, and 30 µm. **b,e,h,** Binary images obtained by the TEM image processing of the panels **a**, **d**, and **g**. **c,f,i,** log(P^2) vs. log(A) plots of Ag filaments printed with 12, 23, and 30 µm.

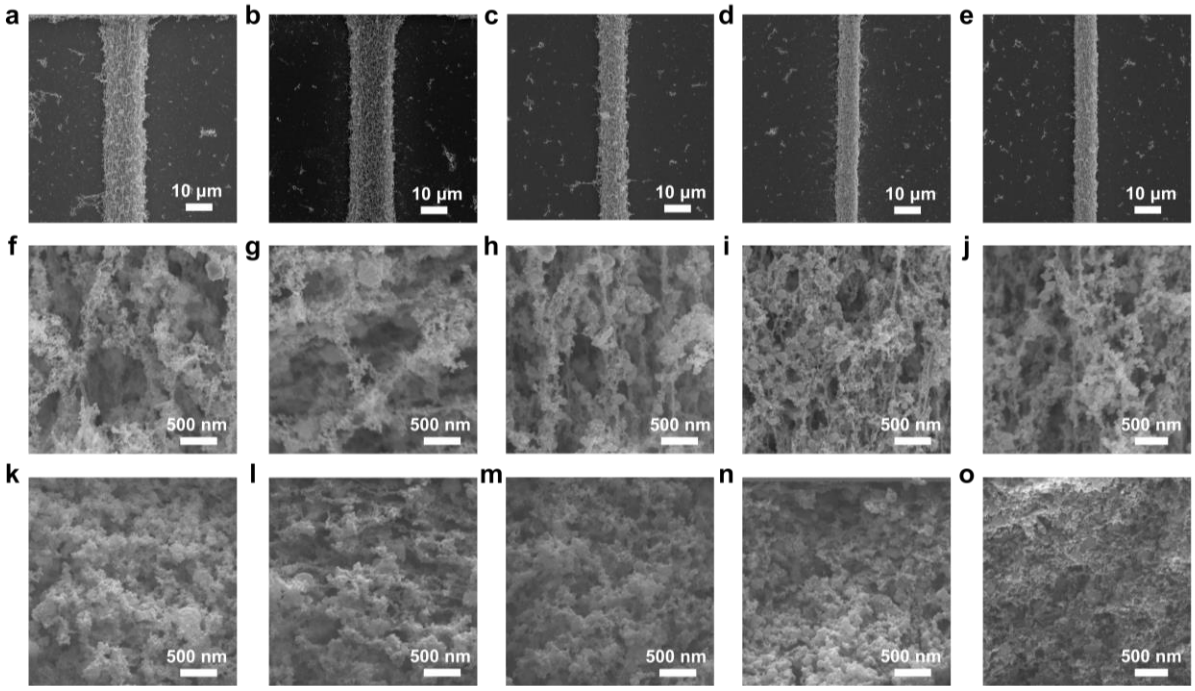

Supplementary Fig. 18 | Microstructure analysis of the printed Ag filaments with varying solvent (NMF)-nonsolvent combinations. a–d, SEM images of Ag filaments printed with ethanol (**a**), propanol (**b**), butanol (**c**), pentanol (**d**), and hexanol (**e**). **f–o,** Top views (**f**-**j**) and cross-sectional views (**k**-**o**) of high-magnification SEM images of Ag filaments printed with ethanol (**f,k**), propanol (**g,l**), butanol (**h,m**), pentanol (**i,n**), and hexanol (**j,o**).

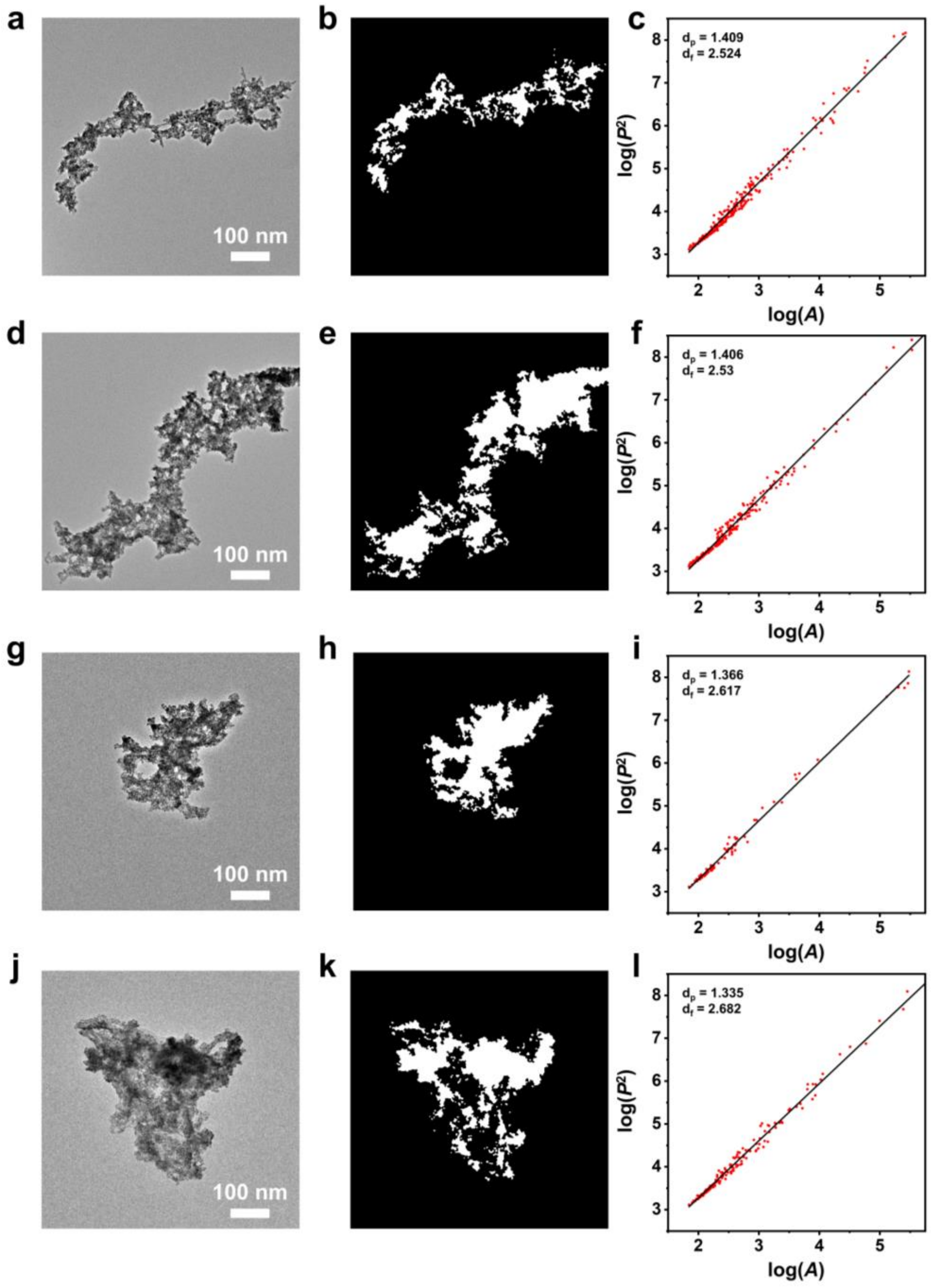

Supplementary Fig. 19 | Fractal dimension calculation of printed Ag filaments with varying solvent (NMF)-nonsolvent combinations. a,d,g,j, TEM images of Ag filaments printed with ethanol (**a**), propanol (**d**), pentanol (**g**), and hexanol (**j**). **b,e,h,k,** Binary images obtained by image processing of Ag filaments printed with ethanol (**b**), propanol (**e**), pentanol (**h**), and hexanol (**k**). **c,f,i,l,** log(P^2) vs. log(A) plots of Ag filaments printed with ethanol (**c**), propanol (**f**), pentanol (**i**), and hexanol (**l**).

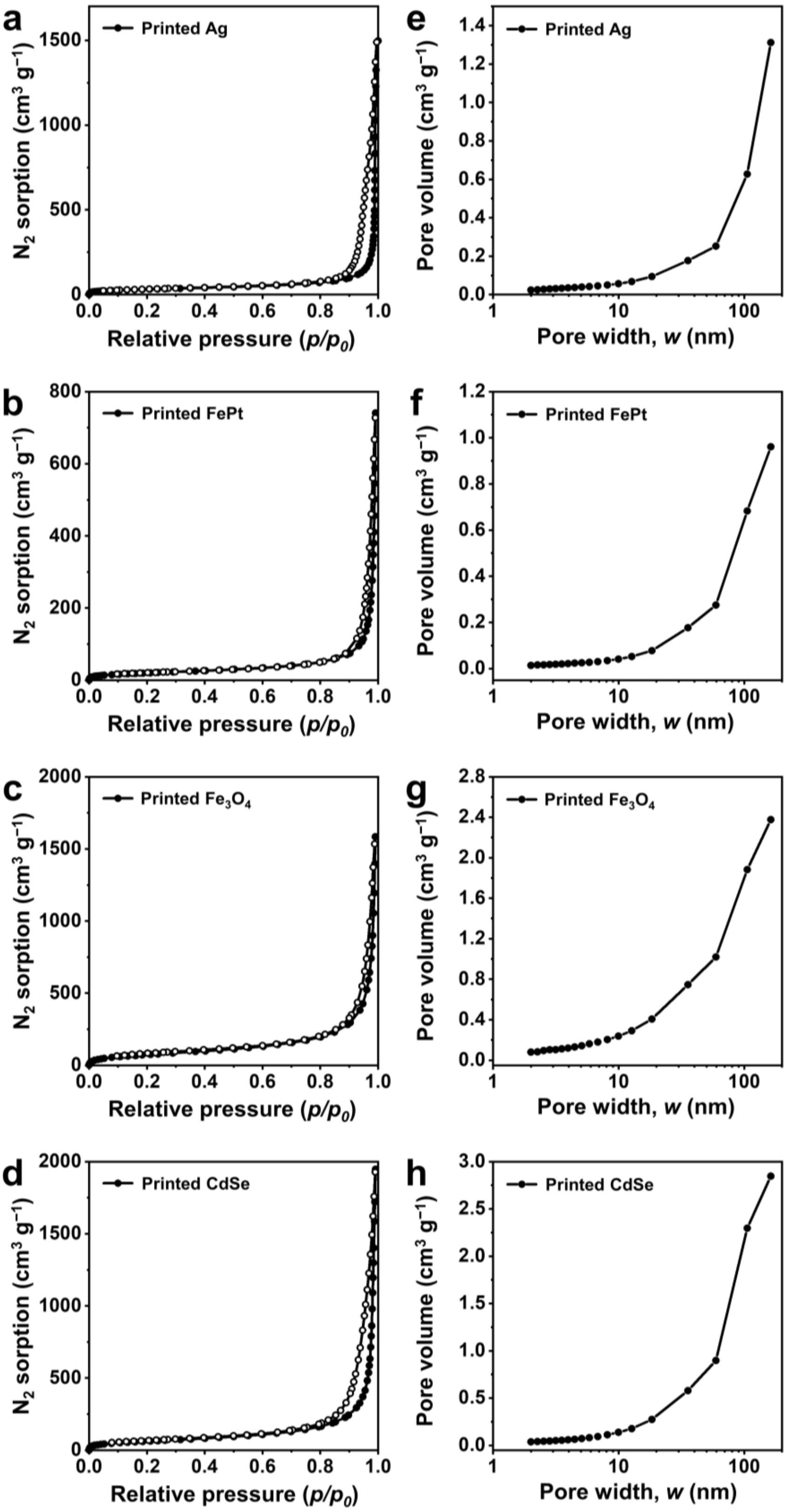

Supplementary Fig. 20 | N_2 physisorption analysis of the printed nanocrystal-based porous materials. a–d, N_2 physisorption isotherms of printed Ag (**a**), FePt (**b**), Fe_3O_4 (**c**), and CdSe (**d**). **e–h,** Pore size distribution of printed Ag (**e**), FePt (**f**), Fe_3O_4 (**g**), and CdSe (**h**).

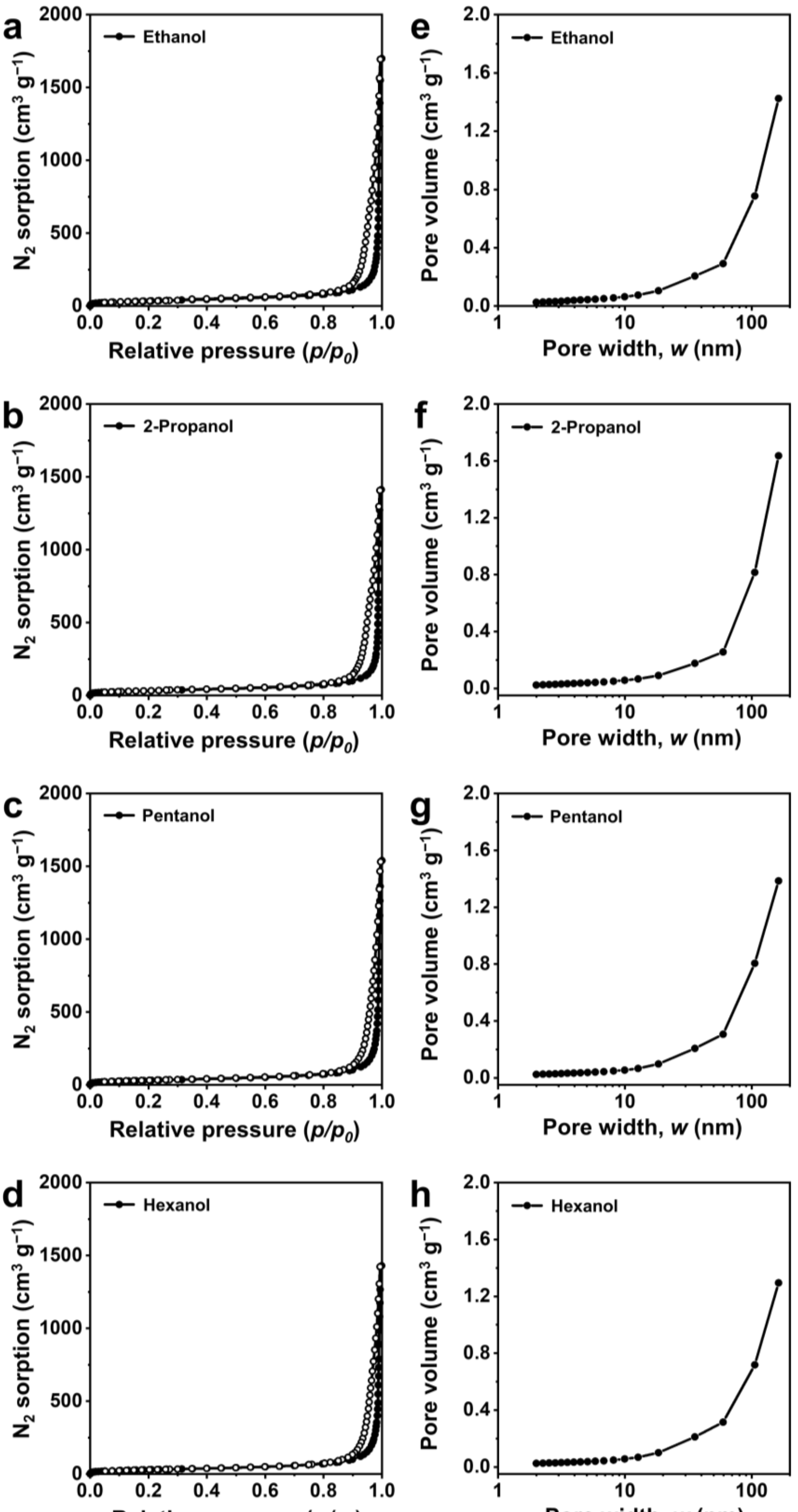

Supplementary Fig. 21 | N_2 physisorption analysis of the printed nanocrystal-based porous materials. a–d, N_2 physisorption isotherms of the printed Ag obtained by the ink deposition in the solidification bath containing nonsolvents of ethanol (**a**), 2-propanol (**b**), 1-pentanol (**c**), and 1-hexanol (**d**). **e–h,** Pore size distribution of the printed Ag obtained by the ink deposition in the solidification bath containing nonsolvents of ethanol (**e**), 2-propanol (**f**), 1-pentanol (**g**), and 1-hexanol (**h**)

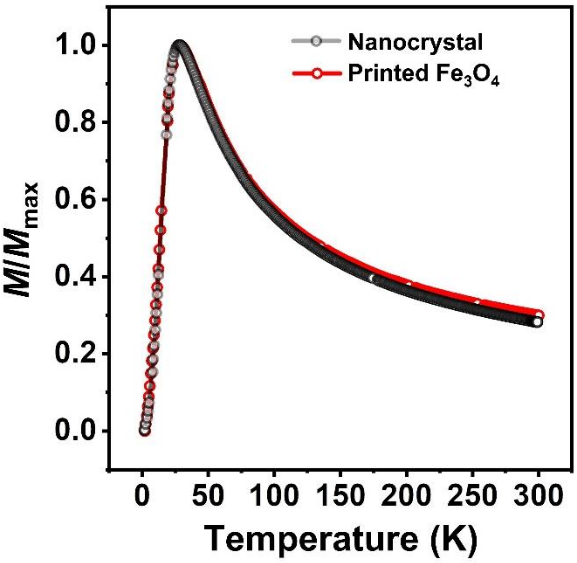

Supplementary Fig. 22 | Magnetic property analysis of the as-synthesized nanocrystal and printed Fe_3O_4 nanocrystal-based porous material. Temperature-dependent zero-field-cooled (ZFC) magnetization of the as-synthesised and printed Fe_3O_4 nanocrystals, respectively.

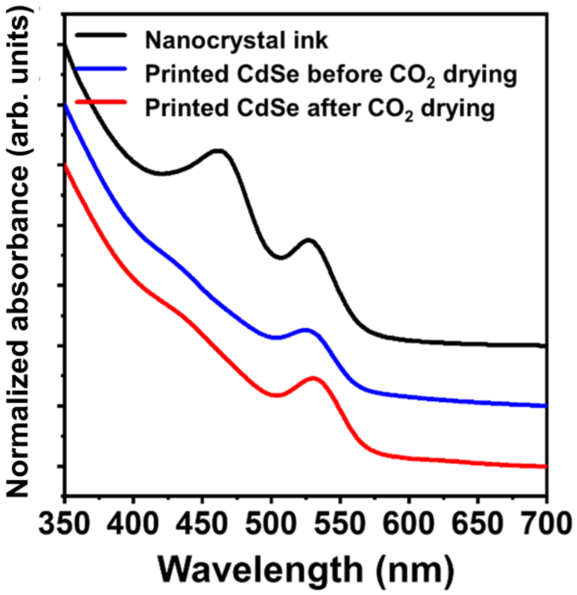

Supplementary Fig. 23 | Optical property analysis of the CdSe nanocrystal ink and printed nanocrystal-based porous material. UV-Vis absorption spectra of the thiomolybdate-capped CdSe nanocrystal ink, and the printed ones linked with Fe^{2+} before and after supercritical CO_2 drying.

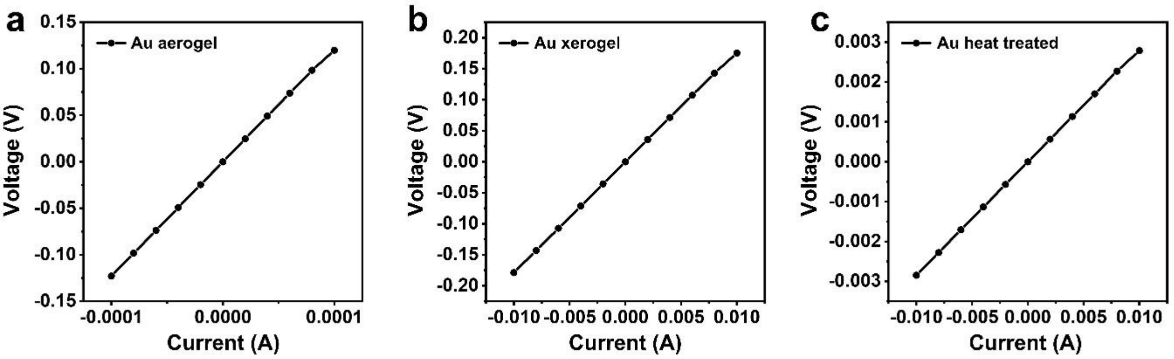

Supplementary Fig. 24 | Electrical property analysis of the printed Au nanocrystal-based porous material. a–c, *I-V* curves of the printed Au aerogel (**a**), xerogel obtained by drying under ambient condition (**b**), and xerogel sample heated at 600 °C (**c**). *I-V* curves were obtained by the van der Pauw method, where a four-point probe placed around the perimeter of the samples were connected to a Keithley 2400 instrument. The Au aerogel, xerogel, and heat-treated samples were prepared as follows. The aerogel was prepared by the supercritical drying of the as-printed thiomolybdate-capped Au nanocrystal in the Au^{3+} linker bath. The xerogel was obtained by drying of the as-printed sample under ambient environment. The heat-treated sample was obtained by the heat-treatment of xerogel sample at 600 °C.

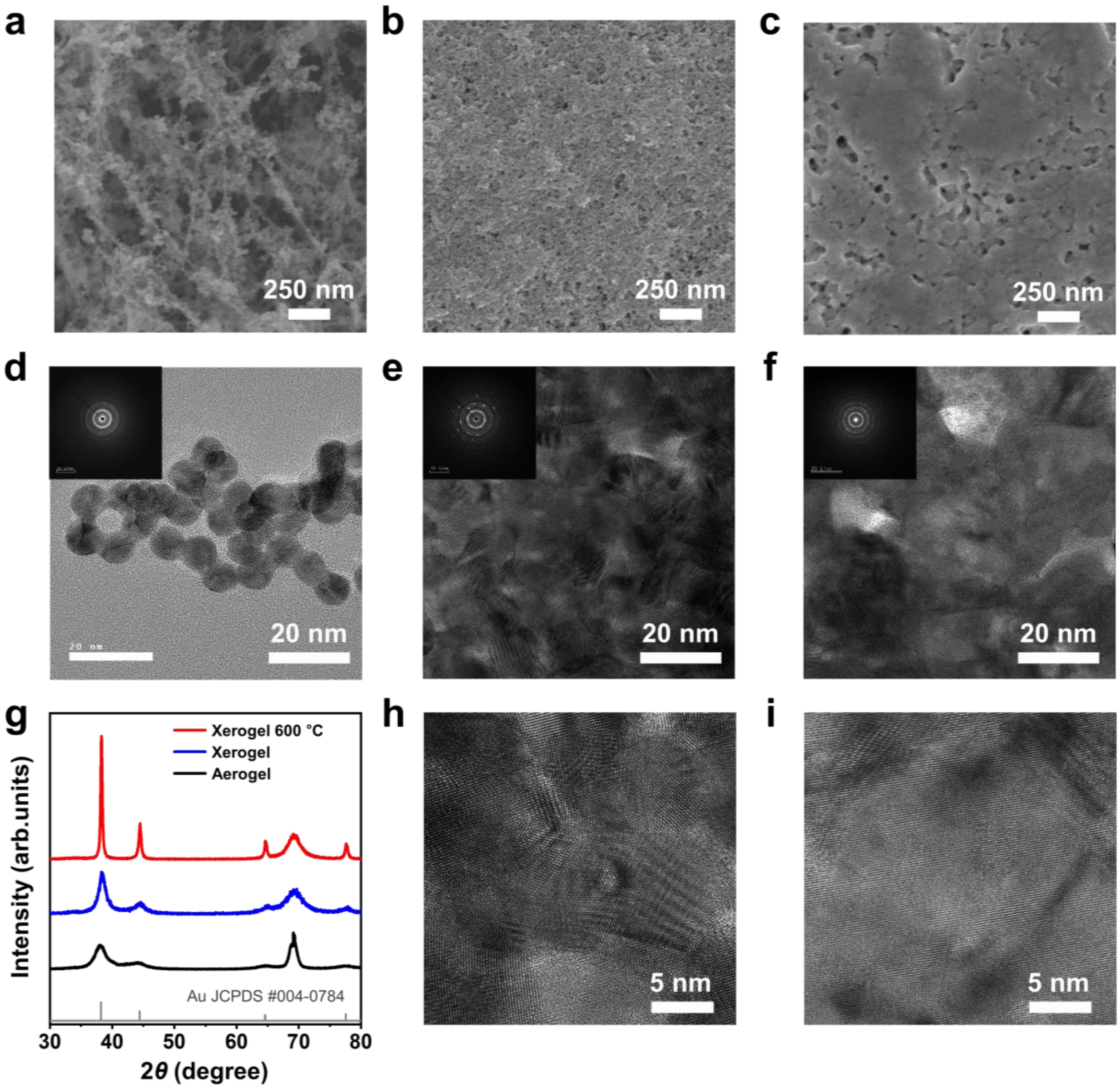

Supplementary Fig. 25 | Microstructure analysis of the printed Au aerogel, xerogel, and heat-treated xerogel. a–c, SEM images of the printed Au aerogel (**a**), xerogel (**b**), and 600 °C heat-treated xerogel (**c**). **d–f,** TEM images of the printed Au aerogel (**d**), xerogel (**e**), and 600 °C heat-treated xerogel (**f**). **g,** XRD patterns of the printed Au aerogel, xerogel, and 600 °C heat-treated xerogel. **h,i,** High resolution TEM images of (**h**) printed Au xerogel and (**i**) 600 °C heat-treated xerogel.

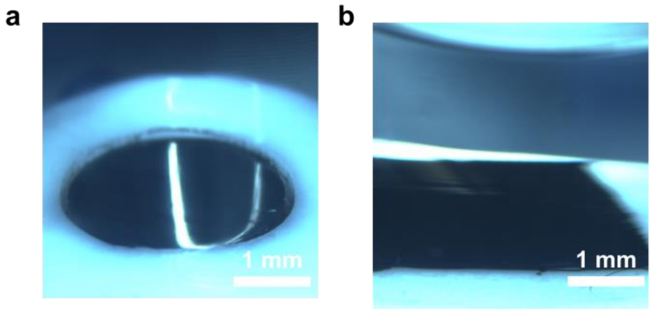

Supplementary Fig. 26 | 3D printing of FePt nanocrystals for the ORR. a,b, CCD camera images of rotating disk electrode (**a**) and FePt nanocrystals directly printed on the rotating disk electrode covering the glassy carbon (**b**).

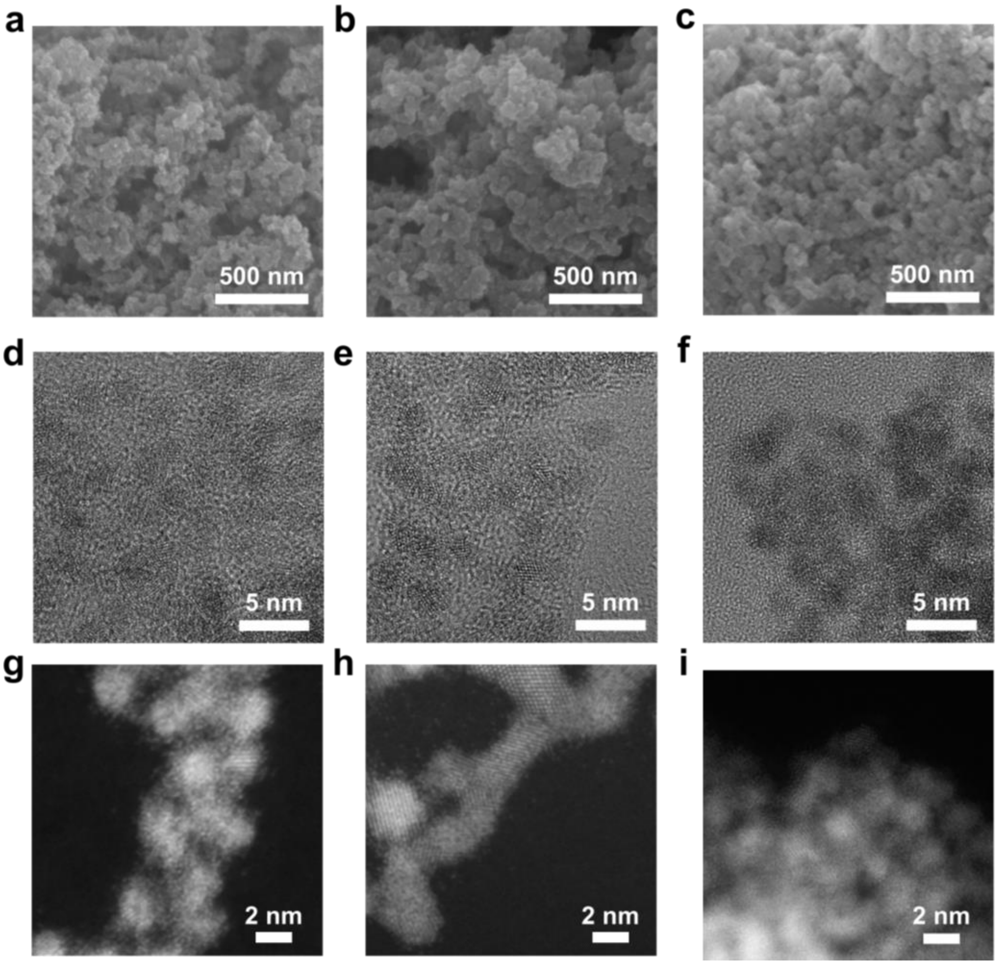

Supplementary Fig. 27 | Structural analysis on the printed and treated FePt nanocrystals. a–i, SEM (**a–c**), HRTEM (**d–f**) and STEM (**g–i**) images of as-printed FePt nanocrystals (**a,d,g**) and the samples after the HCl treatment (**b,e,h**), and subsequent electrochemical activation (**c,f,i**).

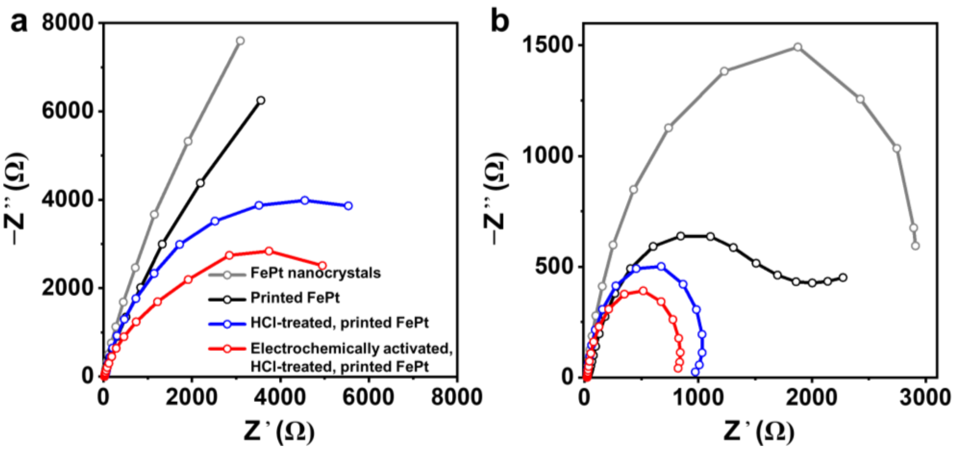

Supplementary Fig. 28 | Electrochemical impedance spectroscopy (EIS) analysis. a,b, EIS analysis of FePt nanocrystal building blocks, as-printed FePt nanocrystal, HCl-treated, printed FePt nanocrystal, and subsequent electrochemically activated sample at 0.9 V vs. RHE in 0.1 M $HClO_4$ saturated with Ar (**a**) and O_2 (**b**).

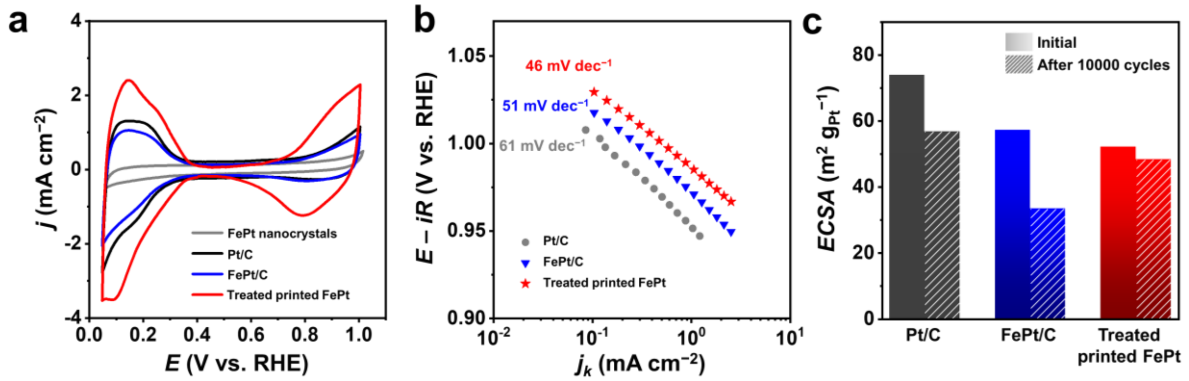

Supplementary Fig. 29 | Electrocatalytic properties for ORR. a, Cyclic voltammogram (CV) of FePt nanocrystal building blocks, Pt/C, FePt/C, and electrochemically activated, HCl-treated, printed FePt nanocrystals (treated printed FePt), The catalyst loading amounts were optimized at 20 μg_{Pt} cm^{-2} for Pt/C and FePt/C and at 50 μg_{Pt} cm^{-2} for treated printed FePt. **b,** ORR Tafel plots of Pt/C, FePt/C, and treated printed FePt. Tafel slopes of each curves are given in the plot. **c,** ECSA values before and after the 10000 cycles of ORR accelerated durability test (ADT) in 0.1 M $HClO_4$.

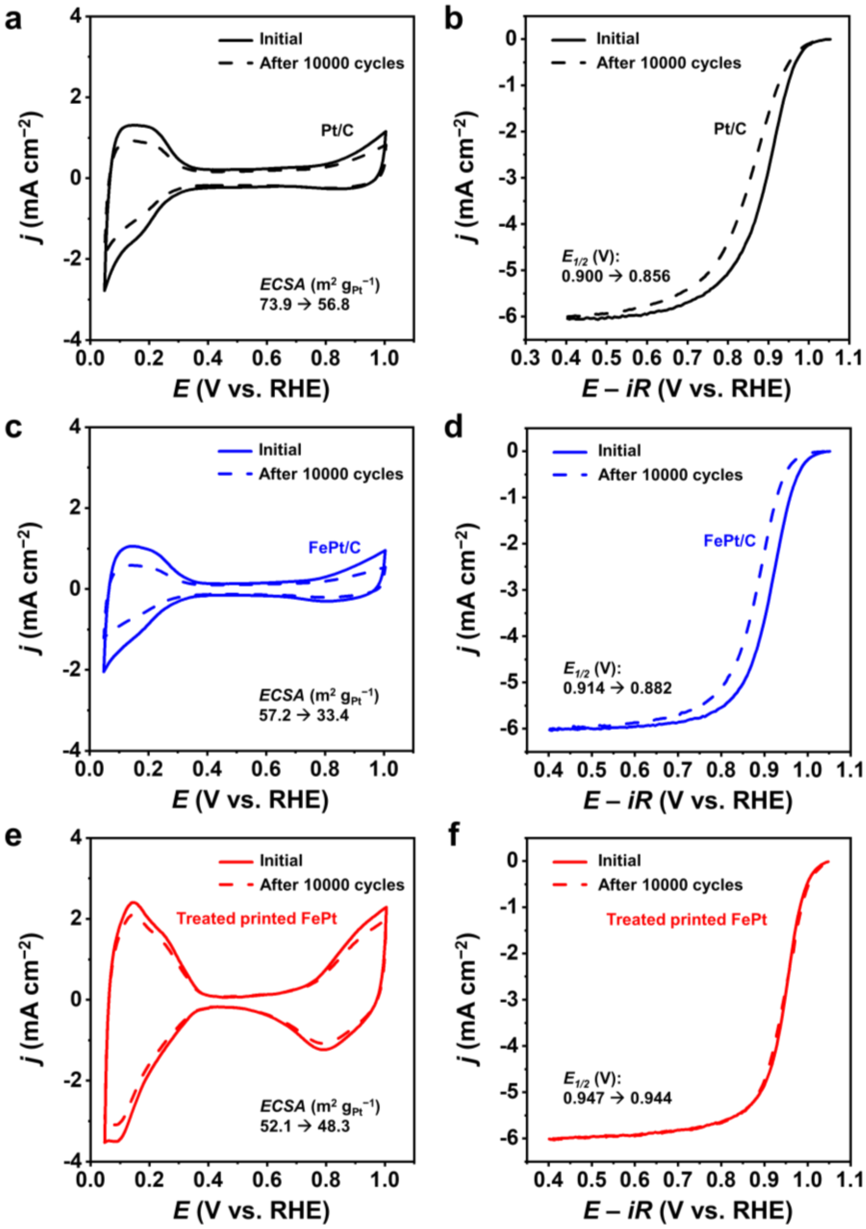

Supplementary Fig. 30 | CVs and ORR curves before and after the ORR ADT. a,c,e, CVs of Pt/C (**a**), FePt/C (**c**), and our treated printed FePt samples (**e**). ECSA values before and after the ADT are given in the plots. **b,d,f,** ORR curves of Pt/C (**b**), FePt/C (**d**), and treated printed FePt (**f**). $E_{1/2}$ values before and after the ADT are given in the plots.

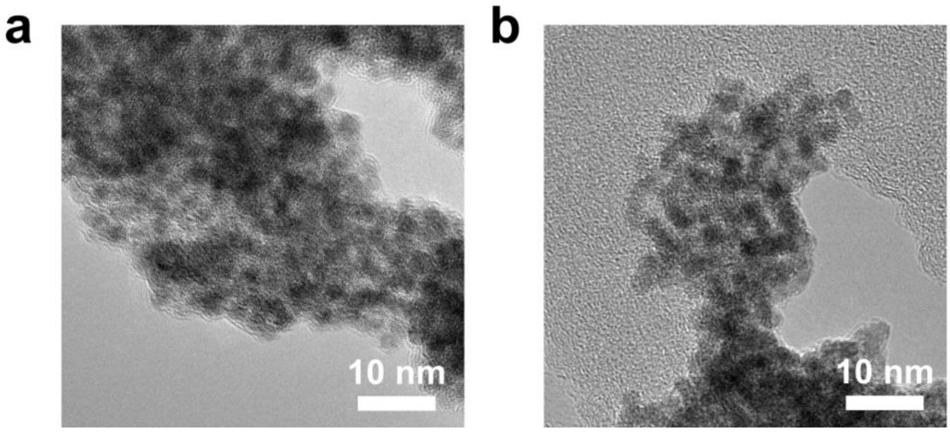

Supplementary Fig. 31 | Microstructural analysis after the ORR ADT. a,b, TEM images of the treated printed FePt samples as coated on the rotating disk electrode (**a**) and after the ORR ADT (**b**).

Supplementary Table 1 | Hansen solubility parameters (HSPs), HSP difference (R_a), and dielectric constant (ε) of solvent and various nonsolvents.

Material	δ_t (MPa)$^{1/2}$	δ_d (MPa)$^{1/2}$	δ_p (MPa)$^{1/2}$	δ_h (MPa)$^{1/2}$	R_a	Dielectric constant (ε)
N-methylformamide[a] (NMF)	30.1	17.4	18.8	15.9	0.0	182
Dimethyl sulfoxide (DMSO)	26.7	18.4	16.4	10.2	6.5	46.7
N,N-Dimethylformamide (DMF)	24.9	17.4	13.7	11.3	6.9	37.7
Acetonitrile	24.4	15.3	18	6.1	10.7	37.5
N-methyl-2-pyrrolidone (NMP)	23.0	18	12.3	7.2	10.9	33
Methanol (MeOH)	29.4	14.7	12.3	22.3	10.6	33.6
Ethanol (EtOH)	26.5	15.8	8.8	19.4	11.1	24.3
Acetone	19.9	15.5	10.4	7	12.8	20.7
Isopropyl alcohol (IPA)	23.6	15.8	6.1	16.4	13.1	18.3
Butanol (BuOH)	23.2	16	5.7	15.8	13.4	17.8
Pentanol	21.6	15.9	4.5	13.9	14.7	15.3
Hexanol	20.8	14.1	8.6	12.7	12.6	13.3
Dichloromethane (DCM)	20.2	18.2	6.3	6.1	16.0	9.08
Tetrahydrofuran (THF)	19.5	16.8	5.7	8	15.3	7.6
Ethyl acetate (EA)	18.2	15.8	5.3	7.2	16.4	6.02
Chloroform	18.9	17.8	3.1	5.7	18.7	4.81
Toluene	18.2	18	1.4	2	22.3	2.4
Cyclohexane	16.8	16.8	0	0.2	24.5	2.02
Octane	15.5	15.5	0	0	31.0	1.95
Hexane	14.9	14.9	0	0	25.1	1.89

[a]Solvent of nanocrystal ink.

Supplementary Table 2 | The calculated nanocrystal sizes from the XRD pattern using the Scherrer formula. The XRD patterns for the calculations were depicted in Fig. 3e and Supplementary Fig. 13.

	**As-synthesised Nanocrystal (nm)**	**Printed nanocrystal (nm)**
Au	4.33	4.93
FePt	1.56	1.21
CdSe	2.37	2.81
Fe_3O_4	4.13	3.97

Supplementary Table 3 | Calculated d_p and d_f of the printed Ag filaments with different diameters.

Diameter (μm)	d_p	d_f
**12**	1.387	2.572
**23**	1.38	2.587
**30**	1.396	2.552

Supplementary Table 4 | Calculated d_p and d_f of the Ag printed filaments utilizing different nonsolvents.

Nonsolvent	d_p	d_f
**Ethanol**	1.409	2.524
**2-propanol**	1.406	2.53
**1-butanol**	1.387	2.572
**1-pentanol**	1.366	2.617
**1-hexanol**	1.335	2.682

Supplementary Table 5 | N_2 physisorption analysis data for the printed nanocrystal-based porous materials.

Nanocrystal	Linker	Nonsolvent	Surface area ($m^2 g^{-1}$)	SiO_2 equivalent surface area ($m^2 g^{-1}$)	Total pore volume ($cm^3 g^{-1}$)
Au	Au^{3+}	BuOH	54.2	430	0.975
Ag	Au^{3+}	BuOH	116	498	1.31
FePt	Pt^{4+}	BuOH	74.3	452	1.04
CdSe	Fe^{2+}	BuOH	226	541	2.88
Fe_3O_4	Fe^{2+}	BuOH	279	596	2.42
Au-CdSe	Au^{3+}	BuOH	132	490	1.55
Ag	Au^{3+}	EtOH	130	558	1.42
Ag	Au^{3+}	2-Propanol	120	515	1.64
Ag	Au^{3+}	Pentanol	116	498	1.39
Ag	Au^{3+}	Hexanol	112	481	1.30

Supplementary Table 6 | Elemental analysis results of the printed Au aerogel, xerogel, and sintered samples obtained from the STEM-EDS analysis. Aerogel, xerogel, and sintered samples were obtained through 3D printing followed by supercritical drying, ambient drying, and heat-treatment of xerogel sample at 600 ºC under H_2 atmosphere, respectively.

Sample	Empirical formula
**Aerogel**	$Au_{0.83}Mo_{0.04}S_{0.13}$
**Xerogel**	$Au_{0.95}Mo_{0.01}S_{0.04}$
**Heat-treated xerogel**	$Au_{0.99}Mo_{0.01}S_{0}$

Supplementary Table 7 | Mo mol% in the as-printed and treated FePt nanocrystals. The as-printed and CO_2-dried MoS_4^{2-}-capped FePt nanocrystals linked with Pt^{4+} was treated 0.1M HCl solution and subsequent electrochemical activation process.

Sample	Mo mol%
**As-printed FePt**	7
**HCl-treated FePt**	4
**HCl-treated and electrochemically activated FePt**	1

Supplementary Table 8 | Activity comparison table of self-supported Pt-based catalysts.

Nr.	Catalyst	PGM loading (μg$_{PGM}$ cm^{-2})	*ECSA* (m^2 g$_{PGM}$$^{-1}$)	E$_{1/2}$ (V)	*SA*$_{0.9V}$ (mA cm$_{PGM}$$^{-2}$)	*SA*$_{0.9V}$ (A mg$_{PGM}$$^{-1}$)	Reference
	**FePt_MoS$_4$_Pt_HCl_Act**	**50**	**52.1**	**0.947**	**0.97**	**0.50**	**This work**
	20 wt% Pt/C (commercial)	20	73.9	0.900	0.41	0.3	This work
r1	PtPd nanotubes	40	15.2	0.84	0.78	0.11	1
r2	PtPdCu nanotubes	20	49.6	0.9	0.68	0.33	2
r3	O-Pt$_3$Co nanowires	55	20.1	0.93	1.56	0.31	3
r4	Meso-PtNi_2	135	15.7	0.9	0.7	0.11	4
r5	Intermetallic FePt nanotubes	70	35.0	0.88	0.41	0.14	5
r6	NiPt 2D nanoframes	35	5.3	0.92	5.80	0.203	6
r7	Mesoporous Pt nanosphere	167	21.6	0.92	0.34	0.07	7
r8	Pt skeletal nanotubes	-	21.1	0.92	0.99	0.21	8
r9	Pt double gyroid thin films	40	38.2	0.87	0.81	0.31	9
r10	Pt membranes	200	40.8	-	0.28	0.16	8

Supplementary Table 9 | Comparison of this work with other additive manufacturing methods for creating 3D porous lattice structures.

Printing method	Materials	Printing resolution	Specific surface area (m^2 g^{-1})	Pore size	Reference	Remark
Digital light processing	Ag, Ni, CuNi, CuNiCoFe, and W-Ni	40 μm	N/A	Determined from printed structure	10	Incorporation of metal precursors into printed hydrogel followed by calcination and reduction process.
Digital light processing	Ni-P, alumina	10–500 μm	N/A	Hollow filament with wall thickness 100 nm–2 μm (Ni-P) 40–210 nm (alumina)	11	Electroless plating of metal or atomic layer deposition of alumina onto printed polymer followed by thermal decomposition of polymer template.
Two-photon lithography	Alumina	2.6 μm	N/A	Hollow filament with wall thickness 100 nm–2 μm (Ni-P)	12	Two-photon lithography of photoresist Au/Ti silicon followed by atomic layer deposition, FIB milling, and O_2 plasma.
Two-photon lithography	Carbon	261–679 nm (98% volume shrinkage)	N/A	Determined from printed structure	13	Two-photon lithography of photopolymers followed by pyrolysis in a vacuum at 900 °C.
Two-photon lithography	Ni-Si	2.6 μm	N/A	Determined from printed structure	14	Two-photon lithography of photopolymers followed by sputtering Ni layer and followed by deposition of Si by PECVD process.
Two-photon lithography	ZnO	250 nm	N/A	Determined from printed structure	15	Two-photon lithography of zinc-ion containing aqueous photoresin and subsequent calcination at 500 °C.
Laser-based nano printing	Quantum dots	77 nm	N/A	Determined from printed structure	16	Light induced desorption of surface thiolate ligands from quantum dots resulting bonding between the particles.
Stereolithography	SiC	250 μm	N/A	Porosity came from building block materials of mesoporous silica nanoparticle. 10–150 nm	17	Stereolithography of graphene-silica composite followed by the pyrolysis.
Direct ink writing	Ag	1-30 μm	N/A	Determined from	18	Direct ink writing of concentrated nanoparticle

				printed structure		inks.
Direct ink writing	Alumina	> 200 μm	N/A	Mesopores<br><br>(23.7 μm) in the filament	19	Foam ink containing bubbles printed and followed by sintering process.
Direct ink writing	Alumina	330-840 μm	N/A	Micro- and mesopores in the filament and macropores determined from printed structure	20	Foam ink containing both alumina and carbon (porogen) particles printed and followed by sintering process.
Direct ink writing	SiOC	250 μm	N/A	Nanoscale porosity within each filament is generated by block copolymer and macropores determined from printed structure	21	Nanoscale porosity is generated within each strut by block copolymer templating followed by photopolymerization and pyrolysis.
Direct ink writing	Yttria stabilized zirconia	1.4 mm	N/A	Determined from printed structure	22	Embedded 3D printing followed by microwave activated curing and subsequent removal of sacrificial support matrix
Direct ink writing	PDMS	5 mm	N/A	Determined from printed structure	23	Rotational multi material 3D printing
**Wet 3D printing**	**Ag, Au, FePt, CdSe, Fe_3O_4, and multi materials**	**7–44 μm**	**54–279**<br><br>**(SiO_2 equivalence**<br><br>**430–596)**	**Micro–macropores in the filament**	**This work**	**Direct solidification of nanocrystals followed by supercritical CO_2 drying process.**

Supplementary Table 10 | Comparison of this work with other additive manufacturing methods for producing microporous or mesoporous materials.

Printing method	Materials	Printing resolution (μm)	Specific surface area ($m^2\ g^{-1}$)	Reference
Direct ink writing	Graphene	400	82	24
Direct ink writing	Graphene/polymer	250	N/A	25
Direct ink writing	Graphene/MnO_2	400	27	26
Direct ink writing	Graphene/ZnV_2O_6@$Co_3V_2O_8$	100–400	800–810	27
Direct ink writing	Graphene/carbon nanotube	250	N/A	28
Direct ink writing	Graphene oxide	100–400	N/A	29
Direct ink writing	Graphene	60–250	212	31
Direct ink writing	Graphene/$Ni_{0.33}Co_{0.66}S_2$	N/A	117	31
Direct ink writing	Graphene/Cu	100	193	32
Direct ink writing	Graphene	250	704–1066	33
Direct ink writing	Graphene/polypyrrole	200	786	34
Direct ink writing	SiO_2	327	751	35
Direct ink writing	SiO_2	840–1200	261–378	36
Direct ink writing	SiO_2-silk fibroin	350	311–798	37
Direct ink writing	SiC	470	N/A	38
Stereolithography	SiC	250	N/A	39
Direct ink writing	Cellulose/polymer	410	N/A	40
Direct ink writing	Cellulose	800	N/A	41
Direct ink writing	Cellulose	300	N/A	42
Direct ink writing	Cellulose	840	N/A	43
Direct ink writing	Au	200	N/A	44
SLA	SiC	250	N/A	45
SLA	PEGDA/CNF	N/A	N/A	46
**Wet 3D printing**	**Ag, Au, FePt, CdSe, Fe_3O_4, and multi materials**	**7–44**	**74–279 (SiO_2 equivalence 326–596)**	**This work**

Supplementary Table 11 | Ink formulation conditions for the printed nanocrystal-based materials.

Printed nanocrystals	Nanocrystal ink concentration (mg mL^{-1})	Linker ions and concentrations (mM)	Nanocrystal ink: linker ion solution volume ratio	Supercritical CO_2 drying time. (hours)
Au	50	Au^{3+} / 1	1:80	3–6
Ag	25	Au^{3+} / 1	1:80	3–6
FePt	45	Pt^{4+} / 1	1:80	3–6
CdSe	50	Fe^{2+} / 1	1:80	3–6
Fe_3O_4	70	Fe^{2+} / 1	1:80	3–6

Supplementary discussion

Microstructural analysis of the printed filaments with different diameters

The comprehensive investigation into the interplay between reaction and diffusion is of utmost importance in enhancing the fundamental understanding of the curing mechanism of nanocrystals in our 3D printing process and facilitating enhanced controllability for multiscale porosity. Accordingly, we conducted the meticulous microstructural analyses on both internal and external structures of three sets of the printed Ag filaments with different diameters with the transmission electron microscopy (TEM), scanning TEM (STEM), and scanning electron microscopy (SEM). To observe the interior of the printed filament, the cross-sectional SEM analysis was conducted on the fractured surfaces of the filaments. The optical microscopy (OM) and low-magnification SEM images (Supplementary Fig. 15a-e) show the printed and CO_2-dried filaments with precisely controlled diameters of 12, 23, and 30 µm. The cross-sectional SEM images of the internal region of the filaments (Supplementary Fig. 15f-h) reveal that all samples exhibited the highly porous feature of interconnected nanocrystal networks throughout the entire region. Notably, no apparent differences in microstructures were observed concerning porosity and pore characteristics among the various samples. Similarly, the outer surface of the filament (Supplementary Fig. 15f-h) displayed microstructures consistent with the corresponding internal structures. To delve deeper into the microstructural characterization, we employed TEM analysis on the cross-sectional sample prepared using a focused ion beam, specifically for the 13 µm filament. We further characterised the microstructures of internal and external regions of the filament with 13 µm by the TEM analysis on the cross-sectional sample prepared using a focused ion beam (Supplementary Fig. 16). TEM images of both the internal and external regions of the filament demonstrated interconnected nanocrystals forming porous networks, with no substantial differences observed between the two regions.

Mass fractal dimensions of the printed filaments with different diameters

To conduct a more in-depth quantitative analysis of the microstructures, we proceeded to calculate the mass fractal dimension (d_f) of the printed filament. The mass fractal dimension is a well-established parameter utilized for characterising fractal structures, including aggregates and agglomerates resulting from colloidal suspensions of particles[47]. Assuming that an aggregate with the gyration radius of R_g consists of N identical particles with the radius of a, the d_f is defined by the following equation,

$$N = k_f \left(\frac{R_g}{a}\right)^{d_f} \quad (1)$$

where k_f is the fractal pre-factor which varies typically between 1 and 1.2, depending on the fractal dimension itself. The aggregates displayed highly diverse fractal structures, influenced by varying aggregation conditions and mechanisms. The interplay of interactive forces, encompassing attractive and repulsive forces, dipolar interaction, and gravity, alongside parameters such as particle concentration, size, and size dispersity, collectively determined the internal fractal structures.

The d_f serves as a valuable quantitative tool for comprehending the formation mechanism of aggregates, particularly in scenarios where the interplay between diffusion and reaction dynamics of particles plays a pivotal role. For instance, in the absence of repulsive forces among particles, they rapidly aggregate upon contact, a phenomenon termed diffusion-limited cluster aggregation (DLCA). Conversely, in the presence of a repulsive potential, only a fraction of collisions leads to particle aggregation, a process referred to as reaction-limited cluster aggregation (RLCA). In these distinct aggregation regimes, the clusters exhibit differing d_f; however, the precise transition values remain elusive owing to the inherent complexity of colloidal systems.

To determine the d_f of the printed filaments with the diameters of 12, 23, and 30 μm, we conducted the TEM image processing for i) the calculation of two-dimensional (2D) fractal dimension (d_p) by plotting area (A) vs perimeter (P) of fractal structures and ii) the calculation of d_f using the relationship between d_f and d_p[48-50] (Supplementary Fig. 17). All TEM images employed for the image processing were included in the revised Supplementary Information and comprehensive computational details are provided in the Method section of the revised manuscript.

The TEM images effectively illustrate the fractal structures of nanocrystal networks in all samples. The computed d_f values for each sample were found to be remarkably similar, ranging from 2.55 to 2.59 (Supplementary Table 3). This observation indicates that the fractal structures within the printed filaments were formed under uniform conditions, independent of their respective diameters. Moreover, the d_f values are in the range of the RLCA. Consequently, it can be inferred that the nanocrystal solidification reactions play a crucial role as the rate-determining step in the solidification of nanocrystals within our printing system, rather than the diffusion of nanocrystals toward each other.

This further implies that the nonsolvent diffusion to nanocrystals upon ink deposition occurs rapidly enough not to impede the curing of the interior of the printed structure. These findings are in alignment with the SEM analysis results, revealing analogous microstructures in both the interior and exterior regions of the filaments, regardless of their diameters. These results strongly suggest the homogeneity of microstructures in the printed filaments, irrespective of their diameters. This homogeneity highlights the reliability and consistency of our 3D printing process, reinforcing the potential for precise control over multiscale porosity in the resulting nanocrystal structures.

Surface treatment and electrocatalytic activity of the printed porous materials

To afford clean surfaces, we removed the excess metal ion linker and surface ligands in printed materials via acid treatment in 0.1 M HCl solution and subsequent electrochemical cycling activation in 1.0–1.5 V (vs. RHE). The degree of the surface cleanliness was evaluated by the elemental analysis to monitor the content of Mo with the Energy dispersive X-ray spectroscopy (EDS) since the MoS_4^{2-} ligand was strongly coordinated to the surface of FePt nanocrystals. As the Pt disintegration is suppressed within this potential range[51], the Mo content was significantly reduced down to 1% (Supplementary Table 7). The scanning electron microscopy (SEM) images after these post-treatments demonstrated the well-preservation of macroscopic porosity in microstructures (Supplementary Fig. 27a-c). The high-resolution transmission electron microscopy (HRTEM) and scanning TEM images reveals that these treatments didn't affect the nanostructural characteristics of materials (Supplementary Fig. 27d-i). The electrochemical impedance spectra (EIS) at 0.9 V (vs. RHE) of the samples showing the smallest semicircle among the Nyquist plots of the electrochemically activated samples also suggested significant improvement in the charge transfer by removal of the inactive surface ligands and linkers blocking the catalytic surfaces (Supplementary Fig. 28).

The impacts of post-treatments were corroborated by the electrocatalytic evaluation[52]. In catalysts' cyclic voltammograms (CVs) (Supplementary Fig. 29a), the as-printed FePt nanocrystals showed only marginal current densities. In contrast, after the two-step treatments of HCl treatment and electrochemical activation, the FePt with clean surface (treated printed FePt) exhibited characteristics of Pt-based catalysts, including adsorption/desorption peaks of H and OH adsorbents. Owing to the well-preserved nanostructures, the clean FePt could highly expose Pt catalytic surfaces, despite the absence of catalyst supports. The electrochemically active surface area (ECSA) of the treated printed FePt self-supported catalysts was 52.1 $m^2\ g_{Pt}^{-1}$, which is comparable to those of commercial carbon-supported Pt nanoparticles (Pt/C; 73.9 m2 g_{Pt}^{-1}), as well as carbon-supported FePt nanoparticles having the same composition (FePt/C; 57.2 m2 g_{Pt}^{-1}). The ORR polarisation curves (Fig. 3k) clearly revealed a much higher ORR activity of the treated printed FePt than that of the as-printed FePt nanocrystals. Notably, it exhibited a higher half-wave potential (E1/2) at 0.947 V than those of FePt/C (0.914 V) and Pt/C (0.900 V). Tafel plots also confirmed that the treated printed FePt exhibited the highest kinetic current density at all potential regions (Supplementary Fig. 29b). The considerably smaller Tafel slope achieved in the treated printed FePt (46 mV dec^{-1}; vs. 51 and 61 mV dec^{-1} for FePt/C and Pt/C, respectively) suggests a much faster ORR kinetics. The ORR mass activity at 0.9 V (vs. RHE) (MA0.9V) of the treated printed FePt (0.50 A mg_{Pt}^{-1}) was greater than those of FePt/C (0.47 A mg_{Pt}^{-1}) and Pt/C (0.30 A mgPt−1), achieving the technical target of US Department of Energy (0.44 A mg_{Pt}^{-1}). Importantly, the ECSA and MA0.9V of the treated printed FePt surpass those of Pt-based self-supported ORR catalysts reported to date (Fig. 3l and Supplementary Table 8).

Moreover, the 3D interconnected contiguous nanostructure of printed materials mitigated the dissolution and agglomeration of individual nanocrystals, enabling excellent ORR durability of the treated printed FePt[53]. After 10000 accelerated durability test (ADT) cycles, the treated printed FePt retained 92.7% of original ECSA, much higher than those of FePt/C (58.4%) and Pt/C (76.9%) (Supplementary Fig. 29c, and 30a, c, e). In the ORR polarisation curves before and after the ADT, they nearly preserved its initial ORR polarization curve, whereas other catalysts underwent significant activity loss (Supplementary Fig. 30b, d, f). Consequently, the treated printed FePt retained 89.8% of its initial MA0.9V, whereas FePt/C (36.7%) and Pt/C (37.2%) showed inferior durability (Fig. 3m). Finally, TEM images after the ADT showed the size and shape of nanocrystals to be virtually identical with those before the ADT, corroborating the excellent durability of our sample (Supplementary Fig. 31).

Supplementary references

1. Chen, Z., Waje M., Wenzhen L. & Yan Y. Supportless Pt and PtPd Nanotubes as Electrocatalysts for Oxygen-Reduction Reactions. *Angew. Chem. Int. Ed.* **46**, 4060-4063 (2007).
2. Zhao, X. *et al.* Octahedral Pd@$Pt_{1.8}$Ni Core-Shell Nanocrystals with Ultrathin PtNi Alloy Shells as Active Catalysts for Oxygen Reduction Reaction. *J. Am. Chem. Soc.* **137**, 2804-2807 (2015).
3. Rößner, L. & Armbrüster M. Electrochemical Energy Conversion on Intermetallic Compounds: A Review. *ACS Catal.* **9**, 2018-2062 (2019).
4. Kim, H. Y. *et al.* Self-supported mesostructured Pt-based bimetallic nanospheres containing an intermetallic phase as ultrastable oxygen reduction electrocatalysts. *Small* **12**, 5347-5353 (2016).
5. Lee, J. *et al.* Development of Highly Stable and Mass Transfer-Enhanced Cathode Catalysts: Support-Free Electrospun Intermetallic FePt Nanotubes for Polymer Electrolyte Membrane Fuel Cells. *Adv. Energy Mater.* **5**, 1402093 (2015).
6. Wang, C. *et al.* Ce-Doped $La_3Si_{6.5}Al_{1.5}N_{9.5}O_{5.5}$, a Rare Highly Efficient Blue-Emitting Phsphor at Short Wavelength toward High Color Rendering White LED Application. *ACS Appl. Mater. Interfaces* **9**, 22665-22675 (2017).
7. Deng, X., Chen, K. & Tüysüz, H. Protocol for the Nanocasting Method: Preparation of Ordered Mesoporous Metal Oxides *Chem. Mater.* **29**, 40-52 (2017).
8. Cheng, N. *et al.* A Fe-doped Ni_3S_2 particlefilm as a high-efficiency robust oxygen evolution electrode with very high current density. *J. Mater. Chem. A.* **3**, 23207 (2015).
9. Kibsgaard, J., Gorlin, Y., Chen, Z. & Jaramillo, T. F. Meso-Structured Platinum Thun Films: Active and Stable Electroctalysts for the Oxygen Reduction Reaction. *J. Am. Chem. Soc.* **134**, 7758-7765 (2012).
10. Saccone, M. A., Gallivan, R. A., Narita, K., Yee, D. W. & Greer, J. R. Additive manufacturing of micro-architected metals via hydrogel infusion. *Nature* **612**, 685-690 (2022).
11. Zheng, X. *et al.* Ultralight, Ultrastiff Mechanical Metamaterials. *Science* **344**, 1373-1377 (2014).
12. Kim, M., Lifson, M. L., Gallivian, R., Greer, J. R. & Kim, B. Enabling durable ultralow-k capacitors with enhanced breakdown strength in denisty-variant nanolattices. *Adv. Mater.* **35**, 2208409 (2022).
13. Zhang, X. *et al.* Lightweight, flaw-tolerant, and ultrastrong nanoarchitected carbon. *Proc. Natl. Acad. Sci. U.S.A.* **116**, 6665-6672 (2019).
14. Xia, X. *et al.* Electrochemically reconfigurable architected materials. *Nature* **573**, 205-213 (2019).

15. Yee, D. *et al.* Additive manufacturing of 3D-architected multifunctional metal oxides. *Adv. Mater.* **31**, 1901345 (2019).

16. Liu, G. *et al.* 3D nanoprinting of semiconductor quantum dots by photoexcitation-induced chemical bonding. *Science.* **377**, 1112-1116 (2022).

17. Guo, Z. *et al.* 3D-printed electrically conductive silicon carbide. *Addit. Manuf.* **59**, 103109 (2022).

18. Ahn, B. Y. *et al.* Omnidirectional printing of flexible, stretchable, and spanning silver microelectrodes. *Science* **323**, 1590-1593 (2009).

19. Muth, J. T., Dixon, P. G., Woish, L., Gibson, L. J. & Lewis, J. A. Architected cellular ceramics with tailored stiffness via direct foam writing. *Proc. Natl. Acad. Sci. U.S.A.* **114**, 1832-1837 (2017).

20. Román-Manso, B., Muth, J., Gibson, L. J., Ruettinger, W. & Lewis, J. A. Hierarchically porous ceramics via direct writing of binary colloidal gel foams. *ACS. Appl. Mater. Interfaces.* **13**, 8976-8984 (2021).

21. Bowen, J. *et al*. Hierarchically porous ceramics via direct writing of preceramic polymer-triblock copolymer inks. *Mater. Today* **58**, 71-79 (2022).

22. Román-Manso, B., Weeks, R. J., Truby, R. L. & Lewis, J. A. Embedded 3D printing of architected ceramics via microwave-activated polymerization. *Adv. Mater.* **35**, 2209270 (2023).

23. Larson, N. M. *et al*. Rotational multimaterial printing of filaments with subvoxel control. *Nature* **613**, 682-688 (2023).

24. Yao, B. *et al.* 3D-Printed Structure Boosts the Kinetics and Intrinsic Capacitance of Pseudocapacitive Graphene Aerogels. *Adv. Mater.* **32**, 1906652 (2020).

25. Guo, F. *et al.* Millisecond Response of Shape Memory Polymer Nanocomposite Aerogel Powered by Stretchable Graphene Framework. *ACS Nano* **13**, 5549-5558 (2019).

26. Yao, B. *et al.* Efficient 3D Printed Pseudocapacitive Electrodes with Ultrahigh MnO2 Loading. *Joule* **3**, 459-470 (2019).

27. Zhao, J. *et al.* Direct Ink Writing of Adjustable Electrochemical Energy Storage Device with High Gravimetric Energy Densities. *Adv. Funct. Mater.* **29**, 1900809 (2019).

28. Guo, F. *et al.* Highly stretchable carbon aerogels. *Nat. Commun.* **9**, 881 (2018).

29. Jiang, Y. *et al.* Direct 3D Printing of Ultralight Graphene Oxide Aerogel Microlattices. *Adv. Funct. Mater.* **28**, 1707024 (2018).

30. Tang, X. *et al.* Generalized 3D Printing of Graphene-Based Mixed-Dimensional Hybrid Aerogels. *ACS Nano* **12**, 3502-3511 (2018).

31. Tang, X. *et al.* Architectured Leaf-Inspired $Ni_{0.33}Co_{0.66}S_2$/Graphene Aerogels via 3D Printing for High-Performance Energy Storage. *Adv. Funct. Mater.* **28**, 1805057 (2018).

32. Rocha, V. G. *et al.* Multimaterial 3D Printing of Graphene-Based Electrodes for Electrochemical Energy Storage Using Thermoresponsive Inks. *ACS Appl. Mater. Interfaces* **9**, 37136-37145 (2017).
33. Zhu, C. *et al.* Highly compressible 3D periodic graphene aerogel microlattices. *Nat. Commun.* **6**, 6962 (2015).
34. Qi, Z. *et al.* 3D-Printed, Superelastic Polypyrrole–Graphene Electrodes with Ultrahigh Areal Capacitance for Electrochemical Energy Storage. *Adv. Mater. Technol.* **3**, 1800053 (2018).
35. Zhao, S. *et al.* Additive manufacturing of silica aerogels. *Nature* **584**, 387-392 (2020).
36. Wang, L. *et al.* Three-Dimensional-Printed Silica Aerogels for Thermal Insulation by Directly Writing Temperature-Induced Solidifiable Inks. *ACS Appl. Mater. Interfaces* **13**, 40964-40975 (2021).
37. Maleki, H., Montes, S., Hayati-Roodbari, N., Putz, F. & Huesing, N. Compressible, Thermally Insulating, and Fire Retardant Aerogels through Self-Assembling Silk Fibroin Biopolymers Inside a Silica Structure—An Approach towards 3D Printing of Aerogels. *ACS Appl. Mater. Interfaces* **10**, 22718-22730 (2018).
38. Guo, P. *et al.* Additive Manufacturing of Resilient SiC Nanowire Aerogels. *ACS Nano* **16**, 6625-6633 (2022).
39. Guo, Z. *et al.* 3D-printed electrically conductive silicon carbide. *Addit. Manuf.* **59**, 103109 (2022).
40. Françon, H. *et al.* Ambient-Dried, 3D-Printable and Electrically Conducting Cellulose Nanofiber Aerogels by Inclusion of Functional Polymers. *Adv. Funct. Mater.* **30**, 1909383 (2020).
41. Li, V. C. F., Mulyadi, A., Dunn, C. K., Deng, Y. & Qi, H. J. Direct Ink Write 3D Printed Cellulose Nanofiber Aerogel Structures with Highly Deformable, Shape Recoverable, and Functionalizable Properties. *ACS Sustain. Chem. Eng.* **6**, 2011-2022 (2018).
42. Håkansson, K. M. O. *et al.* Solidification of 3D Printed Nanofibril Hydrogels into Functional 3D Cellulose Structures. *Adv. Mater. Technol.* **1**, 1600096 (2016).
43. Qian, C. *et al.* All-printed 3D hierarchically structured cellulose aerogel based triboelectric nanogenerator for multi-functional sensors. *Nano Energy* **63**, 103885 (2019).
44. Zhu, C. *et al.* Toward digitally controlled catalyst architectures: Hierarchical nanoporous gold via 3D printing. *Sci. Adv.* **4**, eaas9459 (2018).
45. Guo, Z. *et al.* 3D-printed electrically conductive silicon carbide. *Addit. Manuf.* **59**, 103109 (2022).
46. Sun, D., Liu, W., Tang, A., Guo, F. & Xie, W. A new PEGDA/CNF aerogel-wet hydrogel scaffold fabricated by a two-step method. *Soft Matter* **15**, 8092-8101 (2019).

47. Lazzari, S., Nicould, L., Jaquet, B., Lattuada, M., Morbidelli, M. Fractal-like structures in colloid science. *Adv. Colloid Interface Sci.* **235**, 1-13 (2016).
48. Ferri, G., *et al.* Mass fractal dimension from 2D microscopy images via an aggregation model with variable compactness. *J. Microsc.* **286** 31-41 (2022).
49. Moreaud, M. *et al.* Simulation of large aggregate particles system with a new morphological model. Image Anal. Stereol. **40** 71-84 (2021).
50. Ehrl, L., Soos, M., Lattuada, M. Generation and Geometrical Analysis of Dense Clusters with Variable Fractal Dimension. *J. Phys. Chem. B* **113** 10587-10599 (2009).
51. Reier, T., Oezaslan, M. & Strasser, P. Electrocatalytic Oxygen Evolution Reaction (OER) on Ru, Ir, and Pt Catalysts: A Comparative Study of Nanoparticles and Bulk Materials. *ACS Catal.* **2**, 1765–1772 (2012).
52. Li, M. *et al.* Ultrafine jagged platinum nanowires enable ultrahigh mass activity for the oxygen reduction reaction. *Science* **354**, 1414–1419 (2016).
53. Kim, H. Y. *et al.* Self-supported mesostructured Pt-based bimetallic nanospheres containing an intermetallic phase as ultrastable oxygen reduction electrocatalysts. *Small* **12**, 5347-5353 (2016).
