# Supplementary material for: 3D microprinting of inorganic porous materials by chemical linking-induced solidification of nanocrystals
Source: Nat Commun. 2023 Dec 20;14:8460. doi: 10.1038/s41467-023-44145-7 (PMC10733400; doi:10.1038/s41467-023-44145-7)
Supplement: Supplementary file 3 — Description of Additional Supplementary Files [file 41467_2023_44145_MOESM3_ESM.pdf]

Description of Additional Supplementary Files

File Name: Supplementary Movie 1
Description: 3D microprinting process for Ag cube lattice. The 3D micro-cube lattice was built via the layer-by-layer deposition of 32 Ag filament layers.

File Name: Supplementary Movie 2
Description: 3D microprinting process for CdSe pyramid. The 3D micro-pyramid was built via the layer-by-layer deposition of 36 layers of CdSe squares with different widths.

File Name: Supplementary Movie 3
Description: 3D microprinting process for FePt hexagonal prism. The 3D microhexagonal prism was built via the layer-by-layer deposition of 12 layers of FePt hexagons with different widths.
